# Immunomodulatory Properties of Human Breast Milk: MicroRNA Contents and Potential Epigenetic Effects

**DOI:** 10.3390/biomedicines10061219

**Published:** 2022-05-24

**Authors:** Ma’mon M. Hatmal, Mohammad A. I. Al-Hatamleh, Amin N. Olaimat, Walhan Alshaer, Hanan Hasan, Khaled A. Albakri, Enas Alkhafaji, Nada N. Issa, Murad A. Al-Holy, Salim M. Abderrahman, Atiyeh M. Abdallah, Rohimah Mohamud

**Affiliations:** 1 Department of Medical Laboratory Sciences, Faculty of Applied Medical Sciences, The Hashemite University, P.O. Box 330127, Zarqa 13133, Jordan; nada.nawaf@ymail.com; 2 Department of Immunology, School of Medical Sciences, Universiti Sains Malaysia, Kubang Kerian, Kota Bharu 16150, Malaysia; alhatamleh@student.usm.my; 3 Department of Clinical Nutrition and Dietetics, Faculty of Applied Medical Sciences, The Hashemite University, P.O. Box 330127, Zarqa 13133, Jordan; aminolaimat@hu.edu.jo (A.N.O.); murad@hu.edu.jo (M.A.A.-H.); 4 Cell Therapy Center (CTC), The University of Jordan, Amman 11942, Jordan; walhan.alshaer@ju.edu.jo; 5 Department of Pathology, Microbiology and Forensic Medicine, School of Medicine, The University of Jordan, Amman 11942, Jordan; hananyalu97@gmail.com; 6 Faculty of Medicine, The Hashemite University, P.O. Box 330127, Zarqa 13133, Jordan; khaledalbakri1999@gmail.com; 7 Department of Pharmaceutical Sciences, Faculty of Pharmacy, The University of Jordan, Amman 11942, Jordan; alkhafajienas@gmail.com; 8 Department of Biology and Biotechnology, Faculty of Sciences, The Hashemite University, P.O. Box 330127, Zarqa 13133, Jordan; salim_dr_1954@hotmail.com; 9 Department of Biomedical Sciences, College of Health Sciences, QU Health, Qatar University, Doha 2713, Qatar; aabdallah@qu.edu.qa

**Keywords:** breastfeeding, lactation, epigenetics, miRNA, RNA regulation, DNA methylation, histone modification

## Abstract

Infants who are exclusively breastfed in the first six months of age receive adequate nutrients, achieving optimal immune protection and growth. In addition to the known nutritional components of human breast milk (HBM), i.e., water, carbohydrates, fats and proteins, it is also a rich source of microRNAs, which impact epigenetic mechanisms. This comprehensive work presents an up-to-date overview of the immunomodulatory constituents of HBM, highlighting its content of circulating microRNAs. The epigenetic effects of HBM are discussed, especially those regulated by miRNAs. HBM contains more than 1400 microRNAs. The majority of these microRNAs originate from the lactating gland and are based on the remodeling of cells in the gland during breastfeeding. These miRNAs can affect epigenetic patterns by several mechanisms, including DNA methylation, histone modifications and RNA regulation, which could ultimately result in alterations in gene expressions. Therefore, the unique microRNA profile of HBM, including exosomal microRNAs, is implicated in the regulation of the genes responsible for a variety of immunological and physiological functions, such as *FTO*, *INS*, *IGF1*, *NRF2*, *GLUT1* and *FOXP3* genes. Hence, studying the HBM miRNA composition is important for improving the nutritional approaches for pregnancy and infant’s early life and preventing diseases that could occur in the future. Interestingly, the composition of miRNAs in HBM is affected by multiple factors, including diet, environmental and genetic factors.

## 1. Introduction

Since ancient times, human breast milk (HBM) has been considered the best food for newborn nutrition. Breastfeeding is the process of feeding a young child (under the age of two years) directly from a woman’s breasts during lactation [1]. A report from the World Health Organization (WHO) indicates that feeding for the first six months from mothers’ own milk without any extra external supplements is the optimal nutrition for infants to get benefits for immunity and health outcomes [2]. It is recommended that children continue breastfeeding along with supplemental food until two years of age [1]. Several studies have linked the exclusive breastfeeding duration to protective effects against a wide range of diseases in newborns. These include cardiorespiratory disorders [3], malocclusions [4], pediatric sleep-disordered breathing [5], parent-reported behavioral difficulties [5], gains and losses in weight [6], intelligence and neurocognitive behavior changes [7], type 1 diabetes (T1D) [8] as well as infant mortality [9]. Some civilizations and religions (such as Arab and Islam) use the term “milk kinship” [10,11]. It was shown that individuals breastfeeding from the same woman might cause consanguinity even in cases in which they are not related by genetic background. The consequences of marriage between these individuals are the same as consanguineous marriage and put children born of such marriage at risk for certain disorders [10,12]. Moreover, breastfeeding has many benefits for the physical and emotional health of breastfeeding mothers [13], such as improving uterine involution, reducing bleeding, preventing anemia [14,15], reducing the risk of postpartum depression by regulating cortisol circadian rhythms [16,17], decreasing the risk of type 2 diabetes (T2D), cardiovascular diseases, and metabolic syndrome, and protecting against breast cancer (BC) [18].

Extensive studies have been performed to investigate the composition of HBM. The reviews of these studies concluded that it contains bioactive substances, critical macro and micronutrients, and immune-active factors required to ensure children’s optimal growth; they consider it critical for the early development of neonatal immunity [19,20,21]. More specifically, HBM is mainly composed of around 87–88% water and solid components such as macronutrients. These include nearly 7% (60–70 g/L) carbohydrates that supply energy for growth, body functions and activity, are necessary for providing the building blocks of essential body compounds and shape the metabolic activities of the human gut microbiota. The macronutrients also include 3.8% (35–40 g/L) fats for visual and brain development and 1% (8–10 g/L) proteins as crucial building blocks for growth and development [22,23,24,25].

Moreover, HBM contains essential vitamins for growth, including fat-soluble vitamins such as vitamins A, E and K, as well as water-soluble vitamins such as vitamins C, B2, B3 and B5 [26,27]. HBM is also rich in minerals such as sodium, potassium, chloride, calcium, iron, zinc, copper, magnesium and selenium [28]. These essential minerals are required for many vital mechanisms such as building and maintaining strong bones and teeth, red blood cell production, regulation of immune system function, promoting proper fluid balance, muscle contraction, nerve transmission and protection of cells from oxidative damage [29,30,31,32,33]. Studies have also detected some hormones in HBM, including leptin, adiponectin, gonadotropin-releasing hormone (GnRH), insulin, estrogen, androgens, gastrin, progesterone, resistin and ghrelin [34,35,36,37,38]. Although the roles of these hormones are not completely clear, it is suggested that they are involved in several developmental and growth processes, protecting infants from some diseases (such as obesity) and may forestall the infant’s maturation until they are ready for reproduction [34,35,36,37,38]. Furthermore, HBM contains a variety of compounds with immunological properties (e.g., antimicrobial and antioxidant compounds), viable leukocytes (i.e., macrophages, neutrophils and lymphocytes) and a vast range of soluble and cellular factors (e.g., cytokines, chemokines and nucleotides) to facilitate the development and maturation of immune system and immune functioning [39,40]. HBM contains oligosaccharides (bifidus factor) that are thought to stimulate the growth of more than 200 desirable bacterial strains, the most important being probiotic bacteria, including *Bacteroides* spp., which play a vital function in the early stages of newborns’ gut colonization [41]; *Lactobacillus* spp., which ferment lactic acid bacteria (metabolize lactose and other simple sugars); and *Bifidobacterium* spp., which promote gut barrier function and modulate the immune system response [42].

Unlike other body fluids, HBM is extremely rich in circulating RNA molecules [43]. In the recent two decades, studies have detected a large number of circulating microRNAs (miRNAs) in HBM [44,45,46,47]. Almost 1400 miRNAs have been identified in HBM, and some of them have verified functions in the health and disease of infants [48]. The miRNAs in HBM have emerged as potential immune-regulatory agents targeting immune cells and influencing the development of an infant’s immune system through immune modulation [49,50]. For example, high quantities of miRNAs have been detected in HBM that have potential regulatory effects on the differentiation, maturation, activation and suppression of B and T cells, as well as monocyte development [51,52].

Moreover, miRNAs play a vital role in modulating the expression of a wide range of genes by targeting DNA methyltransferases without permanent changes in the gene sequences [53,54]. This process is called epigenetic regulation, and thus, miRNAs are considered epigenetic modulators. Furthermore, miRNAs affect the protein expressions of the target mRNAs resulting in RNA modifications [55,56], and they may affect histone modifications [57]. Although the epigenetic modifications from HBM miRNA transmission on future generations are not yet fully understood, recent research supports the idea that factors (i.e., miRNAs) modifying the epigenetic mechanisms may be transmitted by HBM and that these epigenetic changes may be transferred transgenerationally [12]. Therefore, studies on the HBM content of miRNAs and their potential epigenetic modifications are of vital importance. This work presents a comprehensive critical review of the immunomodulatory properties of HBM in breastfed infants, with emphasis on the potential roles of milk-derived miRNAs. In this up-to-date review, the available literature regarding miRNAs in HBM are reviewed, and the miRNA–mediated epigenetic regulations are discussed in general and from immunological aspects. This work should be of particular interest to researchers who are investigating HBM miRNAs, as well as to the general public. It may help ensure the quality of future research on epigenetic programming through breastmilk miRNAs.

## 2. The Physiological Basis of Breastfeeding and Milk Composition

**Figure 1:**
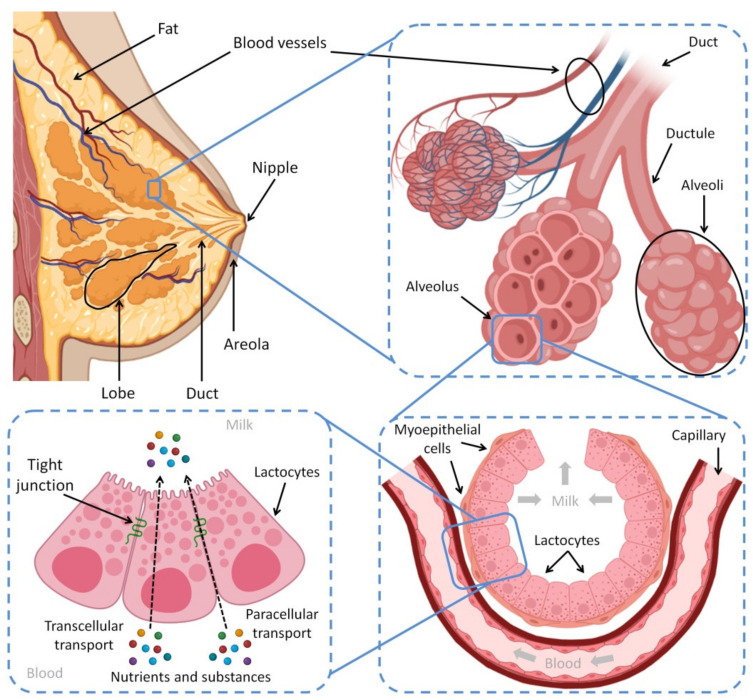
A cross-section scheme of the mammary gland, breast lobe components and process of lactation. Lactation is the process of producing milk from mammary glands in response to hormonal changes, which is secreted in response to an infant sucking. Each mammary gland is composed of a group of alveoli clusters called a lobe, while the alveoli contain balloon-like cavities called alveolus’, which are responsible for milk secretion and storage upon prolactin induction. Alveolus’ are comprised of milk-secreting cuboidal cells called lactocytes surrounded by contractile myoepithelial cells, which in turn respond to oxytocin and push the milk out of the alveoli into the ducts. They also push blood nutrients, immune cells and other molecules across lactocytes into the milk through both the transcellular and paracellular pathways [20,60,61,62]. Created with BioRender.com, accessed on 22 April 2022.

The human breast is composed of mammary tissue, areola, nipple, fat, connective tissues, nerves, lymphatic and blood vessels. The mammary tissue contains the alveoli, which are the milk storage and factory, and the ducts that transport the produced HBM outside the body. The contraction of myoepithelium, muscle cells surrounding the alveoli, compresses the collected milk located inside the alveoli to move through the nine ducts to pass across the nipple—which also has nerves and muscle fibers [58,59]. Furthermore, Montgomery’s glands are present in the circular areola surrounding the nipple [59]. These glands attract the baby to suck from the mother’s breast by secreting a special scent; furthermore, they produce an oily fluid to protect the nipple’s skin during the suckling process [58]. Figure 1 shows the anatomical features of the female breast and the process of lactation.

Oxytocin and prolactin are the most important hormones that have a direct impact on lactation, alongside other hormones like estrogen, which indirectly affect breastfeeding [63]. Suckling the nipple sends sensory impulses to the brain, which stimulates the pituitary gland to secrete oxytocin and prolactin from the posterior and anterior lobes, respectively [64]. The necessity of prolactin lies in the production of milk from the milk-producing cells present in the alveoli. During pregnancy, the blood’s prolactin levels continually increase, which ultimately initiate and control milk production in the mammary tissues [65]. Nevertheless, estrogen and progesterone prevent milk secretion by blocking the activity of prolactin. When their levels reduce after delivery, the secretion of milk begins [66]. Although prolactin is important for producing HBM, there is no longer a strong association between the amounts of milk and prolactin produced [67]. In fact, milk production will eventually stop once a mother stops breastfeeding; the rest of the produced milk will gradually dry up [68].

It is important to know that lactation at night is more helpful in preserving the supply of milk because prolactin production increases at night [69]. Oxytocin stimulates the contraction of myoepithelial cells around the alveoli. It pushes the existing milk inside the lumen of the alveoli to the mouth of the baby and facilitates feeding [70]. The reflex of oxytocin is conditioned to the feelings and sensations of the mother, such as seeing, smelling, and touching the baby. The oxytocin reflex could explain the importance of the baby being close to his or her mother and keeping skin-to-skin contact between them [71,72]. Moreover, lactation is influenced by the emotional changes of the mother; thus, an upset mood inhibits the oxytocin reflex, and otherwise, when the mother gets support and becomes more comfortable, the milk production and letdown will return [73].

## 3. HBM Composition

**Figure 2:**
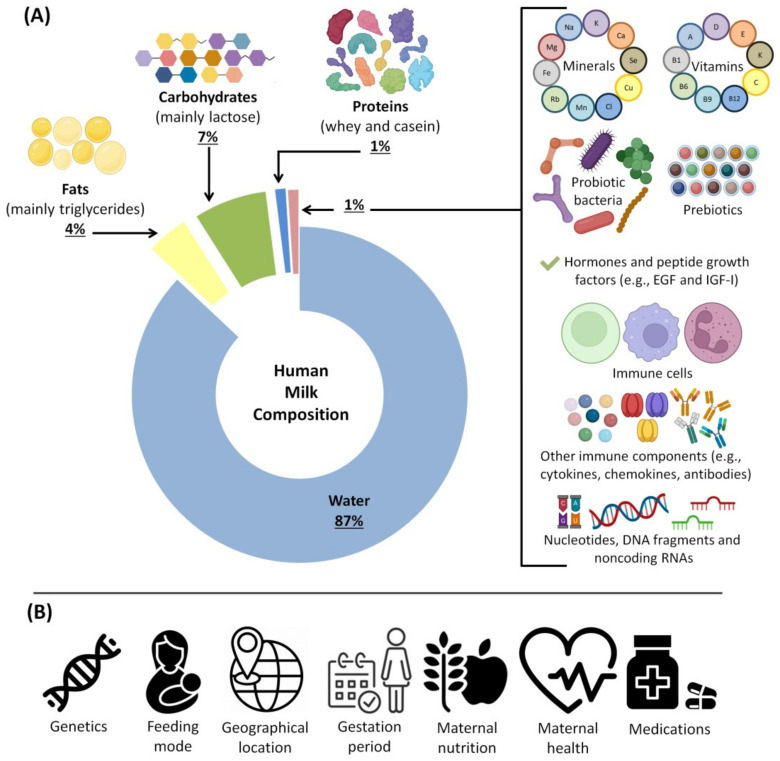
Schematic presentation of HBM components (**A**) and factors affecting its production and composition (**B**). Generally, HBM has four main components, water, carbohydrates, fats and protein. In addition, HBM contains a variety of nutrients, vitamins, minerals, prebiotics, probiotics, hormones, immune cells and substances, nucleotides and nucleic acids and other rare elements. This unique mixture of beneficial components varies due to many factors, mainly related to the mother’s body and health conditions, as well as gestation period [82,83,84,85,86]. Created with BioRender.com, accessed on 22 April 2022.

HBM is the standard for baby nutrition because it is filled with a variety of beneficial substances, including peptide growth factors, hormones [74], prebiotics and probiotics [75], enzymes, cytokines, chemokines, anti-inflammatory elements [76], antimicrobial proteins/peptides [77] and antioxidants [78]. HBM is a person-specific biofluid and includes various bioactive and nutritional components. Bioactive components are the ingredients that affect the body’s condition and function by influencing the biological elements and processes [79]. Different bioactive components are found in milk produced by different sources; some are synthesized by mammary epithelium, some are secreted by cells within the milk, and others come from maternal serum [19,80]. Additionally, milk fat globules secreted from mammary epithelium contain a variety of lipids and proteins [81]. Figure 2 summarizes the beneficial substances of HBM and the factors affecting its composition.

In HBM, fat provides approximately 50% of the total energy, while lactose provides 40% [83]. However, the composition of the milk is influenced by the length of gestation, lactation time, maternal diet, genotype, disease, age of the lactating baby and the onset of the menstrual cycle [87]. For instance, the fat content of preterm milk in early lactation is remarkably higher compared to that of term milk [88]. Furthermore, the average protein concentration gradually declines over the first six months and then stabilizes. Fat is associated with the mother’s diet and has a positive relationship with weight gain during pregnancy. Interestingly, the milk of the mother contains the required components for the development and growth of the infant, even if the mother’s diet is inadequate [83]. Moreover, the amount and nature of HBM components change over time to adapt to the varying needs of the infants’ development [83].

It is important to know the dynamic variability of HBM to reach the maximum benefits. For instance, it is thought that hindmilk enhances premature infants’ growth. It is also rich in lipids, providing the infant with the needs of energy and satiety. Therefore, mothers should completely empty one breast before feeding from the other [19]. Although the fat and protein contents vary regarding the conditions and time, the lactose concentration in mature milk is constant to preserve a stable osmotic pressure in the HBM. The absorption of calcium and other minerals is also mediated by lactose. Lactose attaches to many bioactive carbohydrates like oligosaccharides. Therefore, if there is not enough lactase in the small intestine, lactose malabsorption syndrome will develop. However, it was observed that exclusively breastfed infants rarely have lactase deficiency [83]. Furthermore, to enhance the health and growth of infants, milk components can be adjusted via dietary intake. For instance, maternal diet affects docosahexaenoic acid content. It is reported that 1 g of docosahexaenoic acid supplementation daily in maternal diet augments its level in HBM [89]. Furthermore, mother immunization significantly enhances the protective Igs levels in HBM and protects from influenza infection in both infant and mother [19].

## 4. Breastfeeding and Immunity

In 1891, researchers had shown that immunity was transferred from mother to newborn through breastfeeding [90], and in 1903, this was attributed to the presence of antibodies in mothers’ milk [91]. According to recent findings, HBM contains a rich supply of defensive elements that interact with one another, which serve as an innate immune response that protects against viruses [92,93]. Breastmilk’s innate immune system, in combination with the acquired immune system and intestinal flora, forms a strong component of the gut’s mucosal immunity, which protects breastfed newborns against microbial infections [92,94,95]. The incidence and mortality risks of pneumonia and diarrhea among completely breastfed infants are significantly lower than those who were fed with milk formula [96]. Further, breastfeeding has short- and long-term impacts such as optimal cognitive and behavioral development and protection against allergies and chronic diseases such as hypertension, obesity, diabetes, cardiovascular and autoimmune diseases [97]. Table 1 lists the most abundant immunomodulatory molecules in HBM and their main immunoregulatory functions.

HBM contains two main groups of proteins: whey and casein. These two classes are present with ratios varying from 70/30 to 80/20 and 50/50 in early and late lactation, respectively [131,132]. Lactoferrin, one of the main proteins in the whey class, prevents the spread of bacterial infections among infants. However, there are other available proteins such as α-lactalbumin, cathelicidin-derived antimicrobial peptides and folate-binding protein in HBM [131]. α-lactalbumin is the principal protein found in HBM that is converted in the stomach to “human α-lactalbumin made lethal to tumor cells” (HAMLET). Cathelicidin-derived antimicrobial peptides are produced by HBM cells. They convey protection of the mother from the risk of BC, infection and allergy and protect infants from autoimmune diseases [19]. Furthermore, HBM can act as an inflammatory modulator by suppressing the interleukins participating in the regulation of proinflammatory mediators such as cytokine genes (e.g., *IL-8* gene) [133].

HBM also contains several growth factors that widely impact nervous, vascular, endocrine and intestinal systems [19]. The epidermal growth factor (EGF) is located in the amniotic fluid and has higher levels in the colostrum and preterm milk compared to term milk [134,135]. The EGF stimulates intestinal cells to increase protein synthesis, water and glucose absorption, cell division and DNA synthesis [136]. Further, the EGF is essential for mucosal intestine healing [135]. In addition to IgA, which is the predominant antibody found in HBM [137], stem cell markers estrogen-related receptor beta (ESRRB), cytokeratin 5 (CK5) and myoepithelial marker CK14 are reported as components of HBM [138].

Cytokines are peptides that have many functions and work in both paracrine/autocrine ways [80]. Chemokines are a specific group of cytokines that stimulate the movement of other cells. HBM cytokines are classified into two wide classes: those that protect against pathogens or promote inflammation and those that decrease inflammation [139]. Transforming growth factor-beta (TGF-β) is one of the most common cytokines in HBM [140]. It is activated by the acidity of the stomach. Moreover, TGF-β aids in wound healing and allergic disease prevention [141].

HBM uniquely has a variety of structures and a high concentration of oligosaccharides as prebiotics (non-digestible food ingredients) that enhance the growth of probiotics (microorganisms that afford health benefits to the host when taken adequately) in the intestines [142]. It was reported that formula-fed infants have fewer oligosaccharides than breastfed infants [143]. Additionally, it is well known that breastfed newborns exhibit a high prevalence of the probiotic *Lactobacillus* species, especially *Lactobacillus bifidus*, which acidifies the gut and prevents enteric pathogens from infecting breastfed infants [144,145]. Furthermore, *Bifidobacterium* species are not dominant in the gut of formula-fed infants [146]. Giving probiotics with formula decreases the cases and severity of infant diarrhea [147]. However, most isolated probiotics are taken from fecal infant microbiota or foods [83]. The benefits of prebiotic and probiotic supplementation on infants were investigated by several studies [148,149]. *Clostridium histolyticum* was highly detected in the placebo (control) group compared to the probiotic administered group. Moreover, the prebiotic and probiotic groups were less vulnerable to infection with viral respiratory tract infections. Furthermore, the episodes induced by rhinovirus were significantly higher in the placebo group compared to the probiotics and prebiotics groups [150].

**Figure 3:**
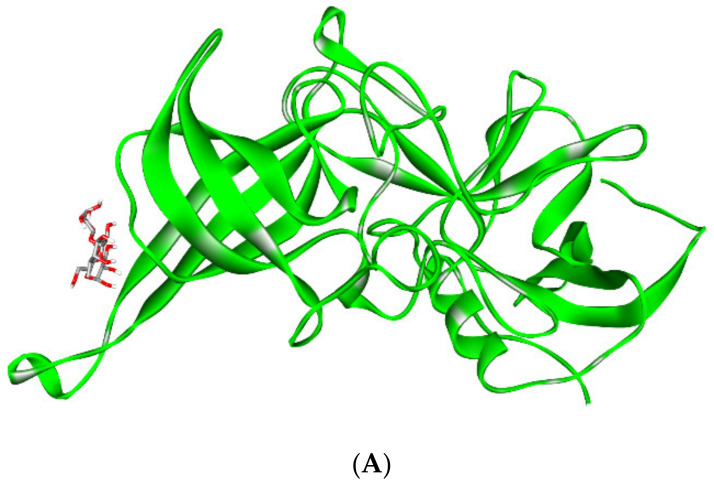

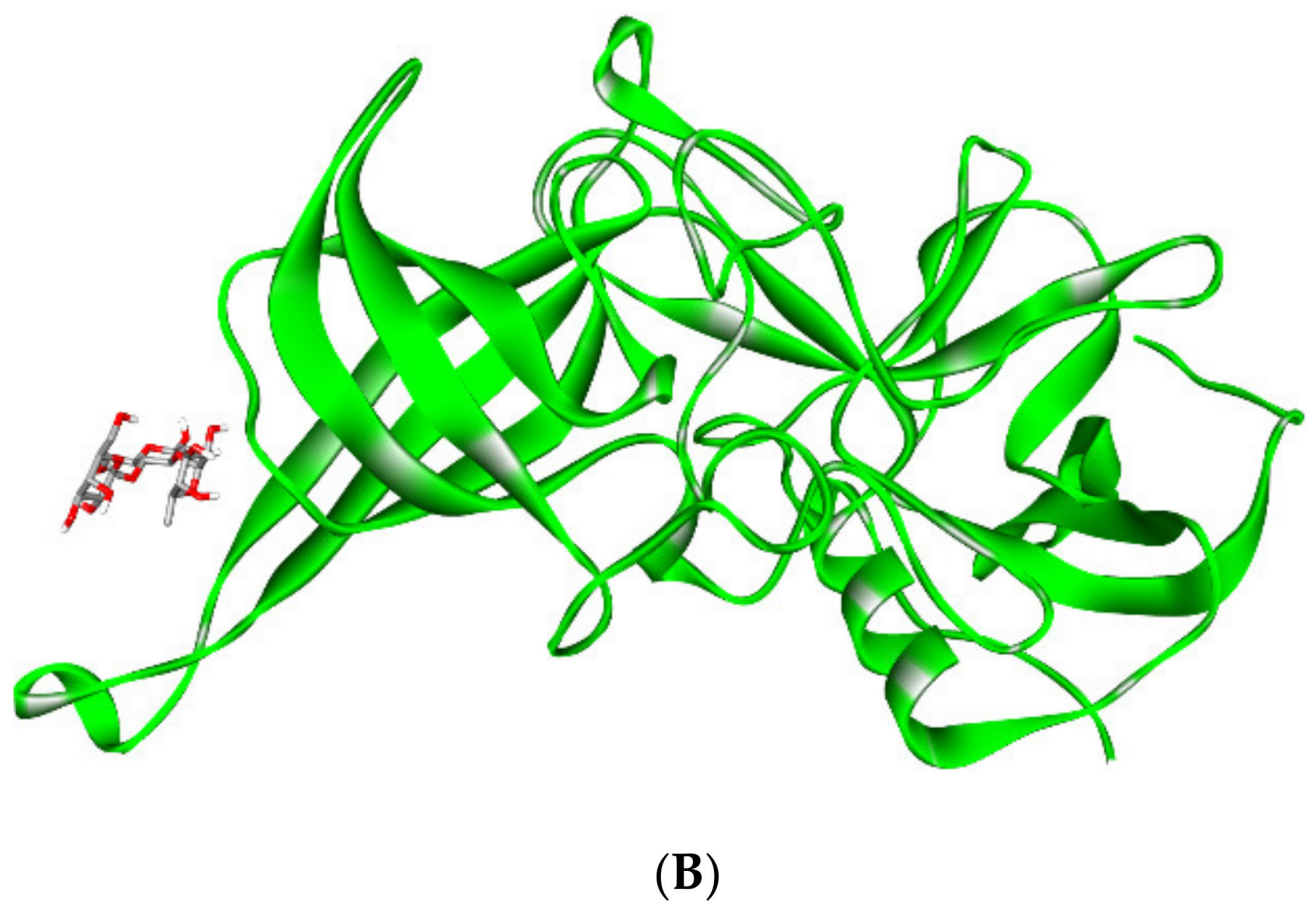
Structural basis for norovirus inhibition by HBM oligosaccharides 2′FL and 3FL. (**A**) Crystal structure of norovirus GII.10 P domain in complex with 2′FL (PDB code: 5hzb). (**B**) Crystal structure of the same domain in complex with 3FL (PDB code: 5hza).

HBM oligosaccharides (HMOs), which mimic histo-blood group antigens (HBGAs) and behave as receptor decoys, interact with noroviruses. It was discovered that an HBM oligosaccharide (i.e., 2′-fucosyllactose (2′FL)) prevents the GI.1 and GII.17 noroviruses from attaching to HBGAs [151]. The results were supported by other studies; two HBM oligosaccharides, 2′FL and 3-fucosyllactose (3FL), have been found to prevent norovirus from binding to surrogate HBGA samples. X-ray crystallography revealed that 2′FL and 3FL bind to the same HBGA pockets on the norovirus capsid, as they structurally resemble HBGAs [152]. These findings show that 2′FL and 3FL may function as natural decoys in humans. Figure 3 illustrates the structural basis for norovirus inhibition by 2′FL and 3FL.

Moreover, catabolic pathways that help the growth of Roseburia and Eubacterium (gut flora linked to protection from immune and metabolic problems and from colorectal cancer) on distinct HBM oligosaccharides were detected [153]. During growth on selected HBM oligosaccharides and in co-cultures with *Akkermansia muciniphila* on mucin, the HBM oligosaccharides pathways were elevated along with additional glycan-utilization loci, suggesting an additional role in permitting cross-feeding and access to mucin O-glycans [153]. Furthermore, *Bifidobacterium longum* subsp. infantis also uses small-mass neutral HBM oligosaccharides, with several of them being fucosylated [154]. A time-dependent effect was discovered in a temporal glycan consumption profile. On the other hand, *Bifidobacterium bifidum* possesses a glycoside hydrolase family (i.e., lacto-N-biosidase) for degrading lacto-N-tetraose and liberating lacto-N-biose I [155]. Overall, this study shows possible symbiosis between humans and bifidobacterial species in the infant gut.

Reactive oxygen species (ROS) are highly oxidizing molecules involved in cellular signaling. Due to their oxidative impacts, high levels of ROS can cause damage to fundamental macromolecular components, including DNA, protein and lipids [156,157]. To override these negative effects, there is an established antioxidant system inside the body [158]. Many antioxidants were found in HBM, such as melatonin, glutathione S-transferase, glutathione peroxidase, catalase, glutathione reductase and superoxide dismutase [159]. They are classified into exogenous and endogenous and further grouped into enzymatic molecules, non-enzymatic molecules and hormones [158]. The antioxidant content is lower in mature milk compared to colostrum, and their activity declines over the breastfeeding period [160]. Regarding enzymatic molecules, catalase—which is composed of four protein subunits—participates in hydrogen peroxide detoxification and helps in ROS elimination [161,162]. Glutathione, one of the main non-enzymatic molecules, regenerates some antioxidants, including vitamin E and C, to their active forms [163]. Melatonin is an endocrine molecule produced by the pineal gland that has protective impacts against aging [164]. Accordingly, it is considered a promising molecule for protecting the nervous system in infants [158].

Overall, breastfeeding decreases the risk for different diseases. Table 2 compares breastfeeding and commercial infant formula feeding to health outcomes. The report was prepared by the Agency for Healthcare Research and Quality (AHRQ) of the US Department of Health Human Services [165,166].

Moreover, morbidity and mortality among breastfed newborns were shown to be several times lower compared to those non-breastfed [167]. This is viewed as a result of the existence of protective chemicals that play a critical role in safeguarding infants’ bodies against diseases, either directly by preventing pathogens from binding to their cellular receptors or indirectly by altering the gut flora [168,169]. Individual differences, mothers’ genotype, infant genotype, concentrations and digestion site are all considered when these bioactive substances act as immunomodulatory molecules [85,170].

## 5. Circulating miRNAs in HBM

**Figure 4:**
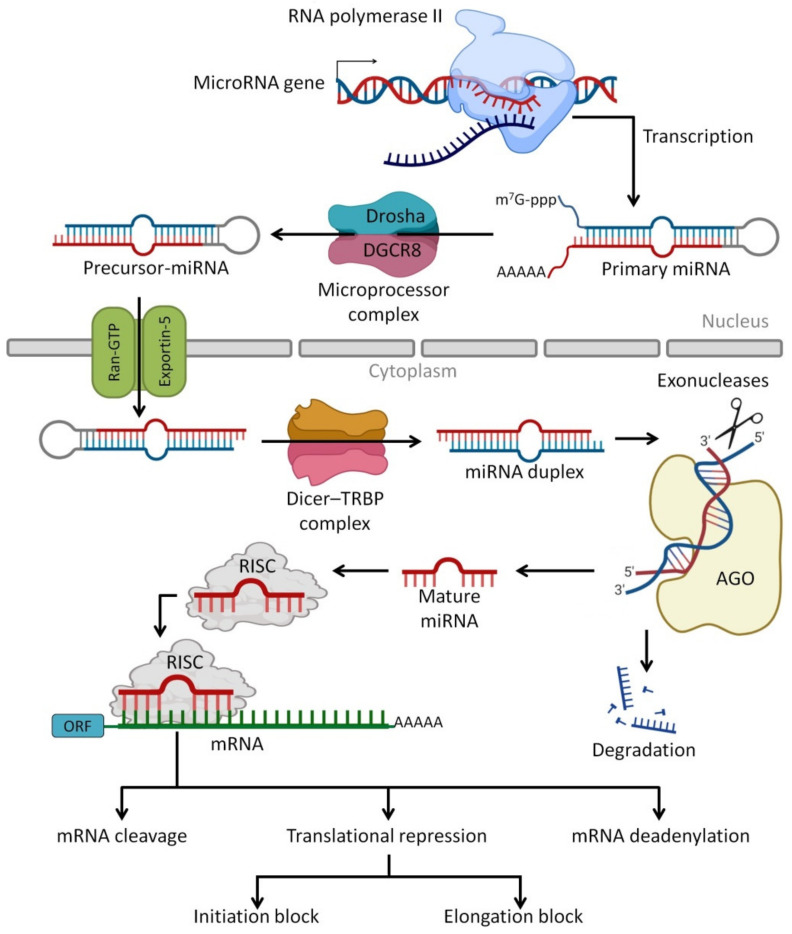
A scheme of miRNA biogenesis and processing pathways. The process starts when RNA polymerase II transcribes the targeted miRNA from DNA sequences into a primary miRNA (pri-miRNA). The RNase enzymes DROSHA and its partner DGCR8 (DiGeorge critical region 8) play a crucial role as a heterotrimeric microprocessor complex by cleavage of pri-miRNA from different sites. The resulting ~70 nt miRNA, called precursor miRNA (pre-miRNA), has a characteristic stem-loop structure and undergoes extensive processing before crossing from nucleus to cytoplasm. The transportation of pre-mRNAs is controlled by exportin-5 (XPO5) in the presence of guanosine triphosphate (GTP)-binding ras-related nuclear protein (RAN). The cytoplasmic pre-miRNAs released through the Ran-GTP/XPO5 complex are triggered by GTP hydrolysis into GDP, which occurs by RAN. In the cytoplasm, the trans-activation response (TAR) RNA-binding protein (TRBP) forms a complex by interacting with the endoribonuclease Dicer, assisting it in finding and cleavage of pre-miRNAs into miRNA duplexes. The duplexes are unwound by binding to Argonaute proteins (AGO), resulting in mature miRNA incorporated into the multiprotein RNA-induced silencing complex (RISC). The miRNAs guide the RISC to bind to complementary regions within targeted mRNA, mediating gene regulation through several post-transcriptional routes, mainly via endonuclease mRNA cleavage or degradation, translation inhibition and deadenylation of mRNA [55,181,182,183,184]. Created with BioRender.com, accessed on 22 April 2022.

miRNAs are the most abundant class of very small regulatory non-coding RNA molecules that are composed of 20 to 24 nucleotides and are capable of controlling 40% to 60% of gene expression at the post-transcriptional level [171,172]. The miRNAs can be produced endogenously, delivered exogenously from neighbor cells as a cell–cell communication, delivered from foods such as plants and human HBM as cell-free miRNAs or via milk exosomes [173]. miRNAs regulate protein synthesis by base-pairing to target mRNAs [174] and lead to suppressed protein synthesis by different mechanisms, including translation repressing or targeted mRNA degradation [175]. Furthermore, there are some miRNAs found in pathways that improve and increase target mRNA expression [176]. In addition to miRNA functions in normal physiological processes such as regulation of gene expression [177], they are utilized as biomarkers for prognosis and diagnosis of cancer, gastrointestinal tract (GIT) disorders, autoimmunity and other diseases [178]. This is due to the presence of some specific tissue-derived miRNAs as extracellular circulating miRNA molecules that are found in body fluids such as plasma, saliva, urine and milk [179,180]. Figure 4 presents the processing pathways of miRNAs in the human body.

miRNAs have been found in higher concentrations in all milk fractions (i.e., cells, lipids and skim) than in other body fluids, including plasma [185]. Milk cells have the largest concentration and variety of miRNAs, while skim milk has the lowest [51,186]. About 1467 recognized miRNAs and 1996 novel miRNAs have been discovered in milk cells [185], while 429 mature miRNAs have been detected in skim milk [187]. In addition, 602 miRNAs were found in isolated exosomes in skim milk [188], and 308 miRNAs were found in milk lipids [189]. Although the substantial heterogeneity of miRNA profiles between different breastfeeding women has been documented [49], the causes for this variability have not been addressed to date, underscoring the importance of future research in this area. A maternal high-fat diet was shown to modulate miRNAs isolated from HBM fat globules, which can modify metabolic pathways in HBM-fed newborns [189].

The miRNA is an important substance in HBM because, firstly, the highest concentration of miRNAs is found in HBM (47,240 μg/L in HBM vs. 308 μg/L in plasma and 94 μg/L in urine) [190], which is attributed to the presence of stem cells in HBM [191,192], and the presence of HBM exosome-derived miRNAs [190]. Secondly, HBM miRNAs are very resistant to harsh conditions such as pasteurization and milk bank storage procedures [49,188], ultraviolet radiation [193], RNase digestion, high temperature, low pH and multiple freeze/thaw cycles [49,188]. Thirdly, high heterogeneity of miRNAs was detected in all fractions of milk [44,190]. Fourthly, the majority of miRNA originates from mammary epithelial cells, with only small fractions originating from maternal blood circulation [44]. That leads to supplying the HBM-fed infants with a wide spectrum of organ-derived miRNAs, such as pancreatic miR-216 and miR-217, hematopoietic-derived miR-142-5 and liver-derived miR-122 [49,188,194,195]. miRNAs in HBM are not limited to endogenous synthesis (either from lactating glands or maternal blood circulation), as food derived-miRNAs have also been detected [196]. This high concentration and heterogeneity of detected miRNAs in HBM exert regulatory functions by targeting a high spectrum of mRNA involved in adaptive and innate immune responses [49], regulating and inhibiting different types of cancers [197], regulating blood lipid profiles [196], regulating blood glucose levels [198] and protecting from cardiovascular disease and sickle cell disease [44].

**Figure 5:**
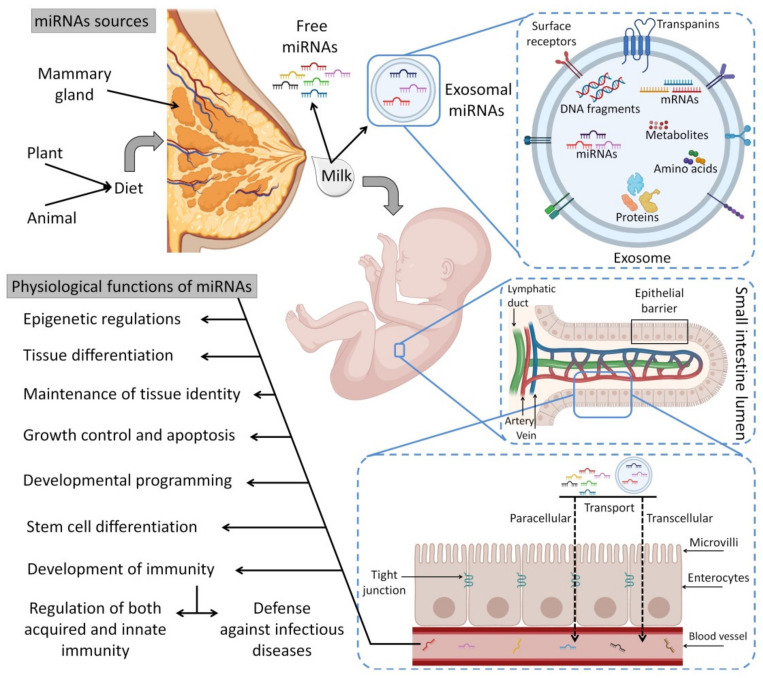
HBM-derived miRNAs and their physiological functions in breastfed infants. Compared to other human biofluids, HBM is an abundant source of miRNAs that present as free molecules or packaged into a type of extracellular vesicle called exosomes. Although the majority of these miRNAs originate from the mammary epithelium, there is a small contribution from the miRNAs that transport from the maternal circulation. Evidence indicates that diet-derived miRNAs from plant and animal sources are presented in human circulation and thus can be transported to HBM. In the infant’s GIT, HBM-derived miRNAs cross intestinal epithelial cells to blood circulation and reach various human organs and tissues. Interactions of miRNAs with their complementary regions within targeted mRNAs affect gene expression and ultimately result in regulating essential physiological functions required for infant growth and development [199,205,206,207]. Created with BioRender.com, accessed on 22 April 2022.

Figure 5 depicts a scenario displaying the sources of exogenous miRNA for the infant (HBM and infant formulas) and their uptake in the infant’s GIT together with other macro/micronutrients (i.e., amino acids and fatty acids). It is worth noting that infant formulae have far lower levels of miRNAs than HBM, with possible changes in their biological activity that need to be investigated further [199,200,201,202,203,204].

### 5.1. Exosomal miRNAs

Exosomes are small membrane vesicles (30 to 150 nm in diameter) produced from endosomes that are discharged into the extracellular environment by several different cells [208]. Exosomes that are found in different physiological fluids (i.e., milk, amniotic fluid, ascites fluid, blood, saliva and urine) have distinct subsets of miRNAs [190]. Exosomes and other extracellular vesicles attach to several categories of cells using endocytosis procedures to transport miRNAs [209,210,211]. Exosomal miRNAs can be protected from degradation in harsh environments [212]. Particularly, milk exosomes protect delivered miRNAs from RNAase digestion, varying pHs along the GIT and cycles of freezing/thawing in the case of frozen HBM [188,199,213,214]. Exosomes allow endocytosis to transport miRNAs from the GIT into the bloodstream and target tissues [215].

Additionally, other microvesicles, including apoptotic bodies (tiny vesicles resulting from the death of apoptotic cells), are also involved in miRNA protection [216]. Furthermore, HBM-derived miRNAs could be completely transported since they are protected within the cells and thus survive the offspring’s GI system and dwell in various organs [56,217]. A few theories have been suggested in relation to free miRNA in milk. RNase can be found in all fluids in the body [218] and degrades RNA molecules into tiny pieces, which indicates its importance in the maturation process of RNA [219]. RNAs, on the other hand, are known to be unstable in harsh environments [220,221]. However, HBM-derived miRNAs remain remarkably stable even when treated with RNase in vitro [222]. Given that milk miRNAs are surrounded by a lipid bi-layered membrane and are supplied with adherence molecules, it has been proposed that their packaging in “vehicle” structures, such as exosomes, somatic cells and other microvesicles, may be vital for their long-distance passage [49,188,223].

### 5.2. Sources of HBM miRNAs and the Effects of Different Conditions

The following factors have an impact on miRNAs in HBM from food sources: (1) foods manufacturing, which may include baking, frying, fermenting and a variety of other processing treatments that may compromise the integrity of small RNA structures; (2) unharmed passage via the GIT since the duodenum contains nuclease enzymes for DNA and RNA; (3) the absorption into the blood via different gastrointestinal barriers; (4) transferring into alveolar cells; and (5) milk secretion by alveolar cells [52,224,225,226]. These five factors contain a variety of chemical environments, some of which cause antagonistic effects against miRNAs. As a result, large miRNA levels should be available in the foods to establish a quantifiable titer in HBM [223]. However, since the neonates lack a well-developed gastrointestinal barrier, larger molecules (such as the mother’s antibodies) may be able to enter directly into the blood circulation. When miRNAs such as species-specific miRNAs are present in the milk, their entrance is enhanced into the bloodstream. Therefore, newborns could be able to pick up miRNAs more easily from food. However, transferring miRNAs from meals into HBM is only possible if they are present in large quantities [49,51,227].

Zhang et al. [228] identified plant food-derived miRNAs in human circulation and bodily fluids, which were tracked by several studies for further investigation to understand their role in mammalian gene regulation [223,225,227,229]. The inability of all plant-derived miRNAs to pass through the GIT into circulation is a key concern when contemplating miRNAs as biological modulators in humans [230], although it has been established that some plant food-derived miRNAs in HBM can target many human mRNAs. For example, miR-156a, miR-166a, miR-167a, miR-172a and miR-168a, target 271, 88, 15, 7 and 4 distinct human mRNAs, respectively [223]. The variety and concentration of human miRNAs may be influenced by factors such as the mothers’ age, body mass index (BMI), neonatal gender, the breastfeeding mother and infant health, term or preterm birth and lactation duration [45,231,232].

According to Carney’s study [46], significant correlations were found between gestational age and 21 of the 26 miRNAs altered in skim or lipid portions in samples of pre-mature infants’ maternal HBM. However, no miRNAs were associated with maternal ethnicity or race, twin pregnancy or maternal hypertension [46]. In either colostrum or mature milk, the intensity of miRNAs was not associated with maternal age at gestational or conception week. Moreover, the contents of miR-378 and miR-30b were higher in colostrum received by girls than in that received by boys. After correcting for maternal pre-pregnancy BMI, this pattern remained for miR-378 [45]. The levels of expression of let-7a, miR-30b and miR-378 were negatively associated with BMI of maternal pre-pregnancy and late pregnancy, but positively associated with maternal weight gain during pregnancy. Furthermore, the level of let-7a in mature milk at the late stage of pregnancy was adversely associated with maternal weight [45].

According to a recent study, there are 63 highly expressed miRNAs in HBM. Of them, 13 are colostrum-specific miRNAs, 13 are mature-specific miRNAs and the rest (37) are common miRNAs [233]. Table 3 lists these miRNAs and extensively discusses their physiological functions in normal and pathological conditions. In addition to the functions listed in Table 3, other studies have confirmed that miRNAs control the expression levels of target genes through synergism, especially knowing that several miRNAs can target 3’UTR of the same mRNA transcript [234,235,236].

### 5.3. Variability in miRNA Expressions in HBM

It has been reported that HBM miRNAs are differentially expressed during lactation stages, for example, the notable drop in the expression level of miR-181a and miR-155 after 6 months of lactation [44]. In a study involving 33 matched samples, the total concentration of miRNA in the fraction of colostrum whey was 87.78 ng/L, which was significantly higher than that in the fraction of mature milk whey (33.15 ng/L). miRNA-378 miRNA-30B and Let-7a were highly expressed in colostrum (4.64, 4.05 and 2.58, respectively) and mature milk (3.62, 4.92 and 2.39, respectively). However, the levels of miRNA-378 and let-7a significantly decreased with the lactation period, while levels of miRNA-30B in mature milk were higher than in colostrum [45].

The change of miRNA content in pre- and post-feeding is a consequence of the change of milk content (such as increase in the cells and fat content) during breastfeeding [590,591], where high content and composition of miRNAs are found in post-feeding [186]; which indicates that breastfeeding enhances the content of miRNAs in HBM. The milk cells and fat contain higher amounts of miRNAs. Those components are elevated in post-feeding due to cell turnover during breast sucking, migration of epithelial cells into milk channels and the process of milk synthesis [590,592]. Unlike miRNAs related to the cell content, elevated miRNAs related to milk fat are significantly correlated with milk volume intake by the infant [186].

Furthermore, in premature infant delivery, an exclusive profile of HBM miRNA with adaptive metabolic targets and functions for growth in premature infants was reported [46]. Several different physiological challenges may occur for premature infants compared to fully mature infants since they require different nutritional needs. There are several significant differences in the expression of 113 miRNAs in skim and lipid samples of mothers of preterm (pMBM) and term infants (tMBM) [46]. The regulation that occurs within the mammary epithelial cell nucleus may play a significant role in the differences in the miRNA composition of pMBM and tMBM. Furthermore, the environmental changes, including abrupt premature delivery, may partially alter the miRNA packaging and extrusion into MBM, which increases the differences in the composition [46]. For example, miRNAs are packaged in several ways, such as shedding, vesicles, RICS–complex protein binding and exosomal transfer [593]. Since each miRNA has a high affinity for specific packaging mechanisms [594], a difference in the carrier ratio may impact the specific secretion of miRNAs in pMBM but not in tMBM and thus increase the differences in macronutrient and micronutrient composition of both milks [88].

Premature delivery, on the other hand, could affect miRNA production within the cell nucleus. The change in maternal hormones could alter the transcription of miRNAs, given the hormonal changes that occur pre- and post-partum. Lactogenic hormones, for example, influence the expression of miRNA and its secretion in cultured mammary cells [165]. Mothers of preterm infants had reduced levels of prolactin [164], which may affect miRNA expression and release. Moreover, the exposure to estrogen hormone changes the content of miRNA in breast cells [595], while the exposure to progesterone changes the processing machinery concentrations of miRNA, which could result in a changed miRNA profile [596]. During pregnancy, the levels of estrogen and progesterone increase; however, lower circulating levels of both hormones in mothers of premature infants at delivery may permanently affect the miRNAs in pMBM and thus enhance the potential evolutionary advantages for the premature neonate, such as influencing glucose homeostasis, regulation of adipogenesis and B-cell proliferation [46].

A high-fat diet during pregnancy alters miRNA expression [597]. Target pathway analysis indicated that changes in miRNA expression due to changes in food consumption might affect the metabolic pathways of either mothers or newborns. High galactose and glucose diets had no significant effect on miRNA species in the milk of mothers [189]. However, the miR-27 and miR-67 expressions were significantly raised under a high-fat diet compared to a high carbohydrate diet [189], which indicated that HBM has a good epigenetic potential in breastfed infants. The changes in the type and quantity of miRNA expression in HBM are considered as a dynamic maternal regulation of infant gene expression based on environmental changes with significant maternal diet distresses. Lactation also enhances the adaptation of mothers and offspring to changes in food supply, which would potentially support an evolutionary advantage for the offspring [598]. Moreover, the impact of both maternal and post-natal diets on modification in the offspring’s hepatic epigenome in animal primate models has been reported [599,600]. It has been found that HBM composition differs with gestational period at delivery and through the first six months of infant age [601]. It is reasonable to suppose that the expression of miRNA would also change during the period of lactation until the weaning of the infant.

## 6. Immunoregulatory Roles of HBM-Derived miRNAs

**Figure 6:**
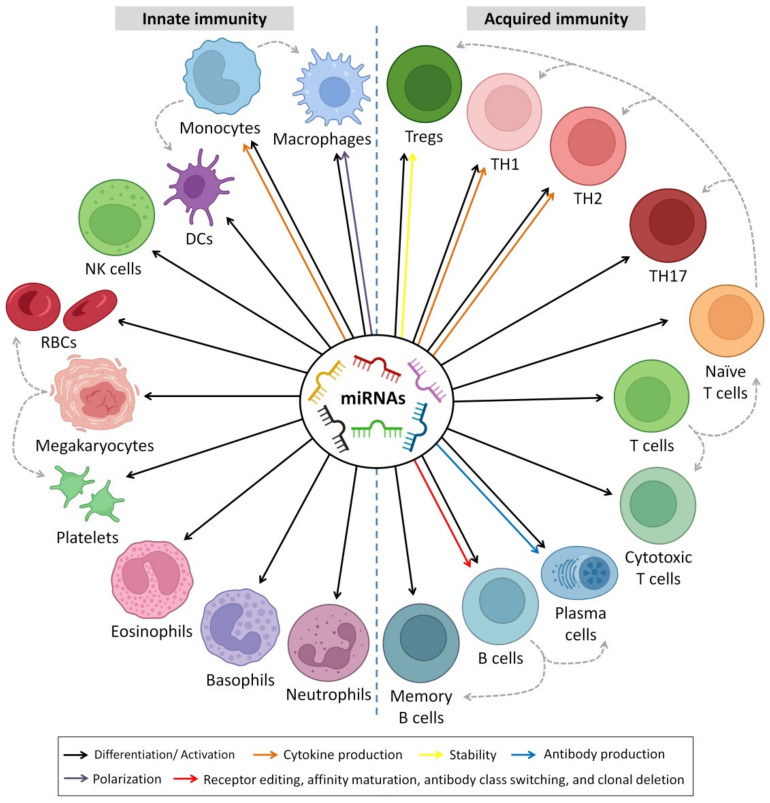
Immunomodulatory actions of HBM-derived miRNAs in both innate and acquired immunity. HBM-derived miRNAs are emerging as key controllers of signaling, differentiation and functions of immune cells, especially T cells. Many miRNAs target cytokine genes in monocytes, T helper type 1 (Th1) and Th2 cells regulating the expression of these cytokines and their circulating levels. Other miRNAs present in HBM have also shown a variety of immunomodulatory actions towards immune cells. For instance, miR-10a is a key regulator of regulatory T cell (T_regs_) specialization and stability. Furthermore, miRNAs not only have the potential to regulate B cell development and functions, but some of them regulate the production of immunoglobulin by plasma cells (e.g., miR-155). Other miRNAs exquisitely regulate receptor editing during B cell maturation (e.g., miR-17∼92 cluster), clonal deletion (e.g., miR-148a), antibody class switching to IgG and secretion of IgE in B cells (e.g., miR-146a). Moreover, HBM-derived miRNAs affect other than-immune system components that participate in innate and adaptive immunity. For example, miR-146 regulates the megakaryocytopoiesis process, which produces platelets and red blood cells (RBCs). miR-27b affects the functions and reactivity pathways of platelets that release inflammatory and bioactive molecules and has some immune functions such as engulfing microbes. Further, miR-142 may affect the survival and functions of RBCs that act as modulators of innate immunity, especially by binding and scavenging specific molecules that mediate inflammatory responses (such as mitochondrial DNA and chemokines) in circulation [51,129,604,605,606,607,608,609,610,611,612,613,614,615]. Created with BioRender.com, accessed on 22 April 2022.

In addition to the biological functions of miRNAs in cell differentiation, metabolism, proliferation, apoptosis, homeostasis and protection from some diseases [602], they are also considered immunoregulatory elements since they play a vital role in differentiation and regulation of the immunological functions in both adaptive and innate immunity [603]. More than 65% of HBM-derived miRNAs are related to immune function [188]. Most miRNAs in HBM are plentiful and known to have immunomodulatory functions (Table 3). These functions are summarized in Figure 6.

HBM-derived miRNAs play a substantial role in the early immune system maturation of infants. Several studies have examined the relationship between miRNAs and innate and adaptive immunological responses [603,616]. According to the Pathway Central database’ annotation (SABiosciences, Frederick, MD, USA), 4 of the top 10 most frequently expressed unique miRNAs (miR-148a-3p, miR-30b-5p, miR-182-5p and miR-200a-3p) are identified as immune-related pre-miRNAs [188]. They showed that milk miRNAs affect T and B cell development [617], neutrophil and monocyte proliferation [618], inflammatory mediators’ secretion [619] and macrophage differentiation [620]. In the mammalian immune system, control of miRNAs has emerged as a fundamental regulatory factor, with any dysregulation leading to immunological disorders and malignancies [3,10,13,621]. For example, the miRNA clusters miR-92 and miR-17 have been found in high concentrations in HBM, indicating their responsibility in the regulation of monocyte development as well as the maturation and differentiation of B and T cells [617,622]. Furthermore, miR-30b-5p increases the cellular invasion and immunosuppression [188,623], and miR-182-5p enhances T cell-mediated immune responses [624], whereas miR-200a-3p is associated with Hodgkin’s lymphoma [625].

MiR-223 is a hematopoietic-specific miRNA that interacts with lineage-specific transcription factors in regulatory signaling networks. In CD34^+^ human hematopoietic progenitors (HPCs) undergoing unilineage differentiation, miR-223 is increased more than 10-fold during granulopoiesis, 3-fold during monocytopoiesis and kept at low levels during erythropoiesis [626]. Perri et al. reported the presence of miR-223 (together with miR-181a) colostrum and HBM and suggested that they operate as selective targets on populations of T cells and granulocytes. As a result, these biomolecules may have an early impact on the immunological homeostasis of newborns. While there was variance in immune-related miRNAs in HBM across breastfeeding women, there was none in colostrum [627]. Furthermore, MiR-223 is thought to play a role in obstructive lung disease as altered expression levels have been observed in both asthma and chronic obstructive pulmonary disease (COPD) [628]. Moreover, miR-223 has been shown to be a potential diagnostic and prognostic marker for many cancers, and it has been reported to suppress osteosarcoma cell proliferation in vitro [629].

Furthermore, HBM contains significant quantities of miR-223, which is believed to trigger granulocyte proliferation [630]. B cell-related miRNAs, such as miR-155 and miR-181, are abundant in HBM [631,632], and they may trigger B cell differentiation. MiR 150, on the other hand, is known to behave as a suppressor of B cells [633,634], despite its lower concentration in HBM. Interestingly, Zhou et al. identified a large number of miRNAs in HBM exosomes [188]. Four miRNAs among the top abundant ten (i.e., miR-182-5p, miRNAs, miR-30b-5p, miR-148a-3p and miR-200a-3p) were linked with immunological processes [188]. MiR-30b-5p, in particular, induces immunosuppression and inhibits activation [623]. In contrast, miR-182-5p stimulates immune responses of T cells [624]. About 59 pre-miRNAs out of 87 (detected in HBM exosomes) showed immunological functions [188]. The miR-17-92 cluster, which was also extremely expressed in HBM exosomes, behaved as a developmental regulator of the immune system [635].

Several miRNA molecules involved in B-cell proliferation pathways, for example, the high expression of the miR-17-92 cluster, are associated with improving B-cell propagation and survival [635]. Furthermore, elevated CD19^+^ B cell expansion was noticed after ectopic high miR-181 levels [636]. In some cases, abnormal elevation of miRNA leads to B-cell tumorigenesis, such as Hodgkin’s lymphoma [637], Burkitt lymphoma [638] and lymphoblastic leukemia [639]. The regulatory mechanisms of miRNAs to B-cells are not limited to enhancing cell proliferation and survival; some miRNAs exert normal controlling functions when expressed at low levels. For example, miRNA-150 interferes with the nuclear transcription factor gene *c-Myb,* which involves B-cell differentiation [633,634]. The first study to understand the role of miRNA in B-lymphocyte differentiation stages was on the protein-coding gene *argonaute RISC catalytic component 2* (*AGO2*), which leads to cells stuck at the pre-B-cell stage and failure of successful progression to mature B lymphocytes [622]. The *AGO2* is important to the synthesis and functioning of miRNAs in hematopoietic stem cells *AGO2* [622,640]. Moreover, the study showed that the *Dicer* gene deletion, an important gene for RNA interference molecules biogenesis, led to defects in B-lymphocyte differentiation, programmed cell apoptosis and antibody production [622].

Early T progenitor cells, with the deletion of the *Dicer* gene, led to a massive decrease in mature T cells without altering the expression patterns of CD8 and CD4 markers during T cell maturation [641]. Whereas deletion of the *Dicer* gene in the single-positive stage led to less reduction in T cell count in comparison with deletion at early stages [642]. miRNA-181 is involved in signal transduction during T cell differentiation and subsequently enhances positive and negative selection [643], sensitizing the T cell receptor to stimuli [643] and reaching hemostasis in cases of over-expression of T cells [635]. MiR-101 regulates the post-transcription of CD278; abnormal alteration of miRNA-101 leads to autoimmunity disease by the production of effector T cell (T_eff_) phenotype [644]. Targeting miRNA-155 to the protein-coding gene *suppressor of cytokine signaling 1* (*SOCS1*) improves the response of regulatory T-lymphocytes to IL-2, which enhances cell survival [645].

In addition to the role of miRNAs in adaptive immune response, miRNAs are involved in several mechanisms in the innate immune response. miRNA-223 controls granulocytic differentiation and granulopoiesis [646]. Induced ablation of miRNA-223 leads to an elevated number of granulocyte progenitors and neutrophil hyperactivity, which leads to spontaneously developing inflammatory and exaggerated tissue destruction [630]. MiRNA-125 interferes with tumor necrosis factor-*ɑ* (*TNF-ɑ*) gene; therefore, a low expression level of miRNA-125 is required to establish a macrophage-mediated inflammatory response [647]. It has been reported that miR-146b-5p targets NF-κB signaling in innate immune responses [648]. The interplay of miRNA action mechanisms and their effect on downstream gene expression is not clear, especially those genes involved in innate immunity [649]. MiRNA-155, on the other hand, is found in significant abundance in HBM and has a regulatory role in cellular (B and T cells) and innate immune response. Moreover, some miRNAs may have roles in reshaping immune responses against microbial infections [650]. For example, it has been reported that miR-29a-3p can suppress the immune responses to intracellular pathogens by targeting IFN-γ [651].

Toll-like receptors (TLRs) are proteins that show a crucial function in the innate immune and digestive systems [652]. TLRs are a large collection of receptors that range from TLR1 to TLR13 [653,654]. HBM also inhibits the TLR signaling pathways of the intestinal epithelial cells, lowering the risk of enteric inflammation [102]. The presence of TLR regulatory components in HBM promotes the use of safe oral prophylactic and therapeutic treatments for inflammatory bowel disease and other gastrointestinal inflammatory disorders caused by aberrant TLR signaling. This was shown by inflammation suppression in rat gut models by using HBM [122]. It was found that miRNAs have a significant role in modulating TLRs; for example, the miR-146 (present in HBM) targets Traf6 and Irak1, components of the TLR signaling pathway activated by LPS, suggesting a negative feedback loop [655]. This field of research is still immature, and extensive investigations are needed to solve the mysteries behind the effects of breastfeeding, as these studies may be useful for manufacturing additives for formulas.

Furthermore, miRNA can influence the development or prevention of autoimmune disorders such as inflammatory bowel disease (IBD) and play a significant role in their development or prevention [49,656]. miRNAs could potentially be applied as biomarkers of milk to identify disorders in the immune system, including allergic illnesses [657,658]. Kosaka et al. [49] found high levels of miRNAs in HBM during the first 6 months of lactation with immune system functions; these miRNAs include miR-150, miR-181a, miR-155, miR-17 and miR-223. MiR-155 and miR-181, which are the most common in the control of B cell variation [607,635], were found in high concentrations in HBM [49,189], implying that they have a role in the immune system development of the infant.

## 7. Breastfeeding and Epigenetics

Epigenetics reflect all molecular mechanisms transforming the expression of genotype into phenotype [659]. This occurs by covalent modifications of DNA by methylation of cytosine [660], mainly at CpG dinucleotides [661]; adenine and guanine methylation [660]; or histone protein modification by deacetylation, methylation or phosphorylation [662], which regulates gene expression by chromatin remodeling [663]. The human epigenome functions as a connection between the inheritable genetic information of humans and its response to environmental factors. Furthermore, variations in human epigenome patterns have a key role in individual response and susceptibility to future toxicant exposure and consequent disease outcomes. Moreover, the epigenetic system consists of nuclear information, which is heritable during cell division and is responsible for controlling cell development, cellular responsiveness and tissue differentiation [664].

### 7.1. MiRNAs–Mediated Epigenetics and Immunity

Over the last few years, many researchers have reported interesting associations between a number of miRNAs with epigenetic alterations leading to the occurrence of various diseases such as cancers [665]. Furthermore, it has been shown that miRNAs are controlled by epigenetic mechanisms; they were also shown to have a reciprocal role in epigenetic regulations. Some of the miRNAs are called epigenetic-miRNAs (epi-miRNAs) as they can control and regulate the epigenetic enzymes and regulators [666,667]. On the other hand, epigenetic enzymes can control the expression level of tumor suppressor miRNAs, and vice versa, these enzymes can also be regulated by reverse responses of the targeted miRNA. Thus, since epi-miRNAs can affect gene expression, many studies showed that epi-miRNA could serve as a fascinating therapeutic tool for diseases initiated by epigenetic dysregulation, such as cancers [665,668].

It was shown that miRNAs could regulate and affect epigenetic DNA-methylation by targeting DNA methylation enzymes (DNA methyltransferases; DNMTs). DNMT-3A and B serve as targets for miRNA; miRNA-29 family members were the first discovered as epi-miRNAs due to their direct influences on DNMT-3A and B in lung cancer [666]. In this study, they found that the miRNA-29 family, with its subtypes 29a, 29b and 29c, has base pairing complementary to the 3′ end of UTRs in DNMT-3A and B. Thus, the expression of miR-29s led to decreased DNMT3A and B expression in lung cancer, supporting the vital role of miR-29s as epigenetic regulators. Furthermore, another study showed that the miRNA-290 family is mainly found in mammalian placenta, which directly targets and regulates the expression of post-transcriptional factor *Rbl2* gene, which acts as a repressor for DNMT 3A and 3B leading to hypomethylation [669]. Thus, miRNA-290 indirectly acts as a regulator for DNA methylation via targeting transcriptional factors.

**Figure 7:**
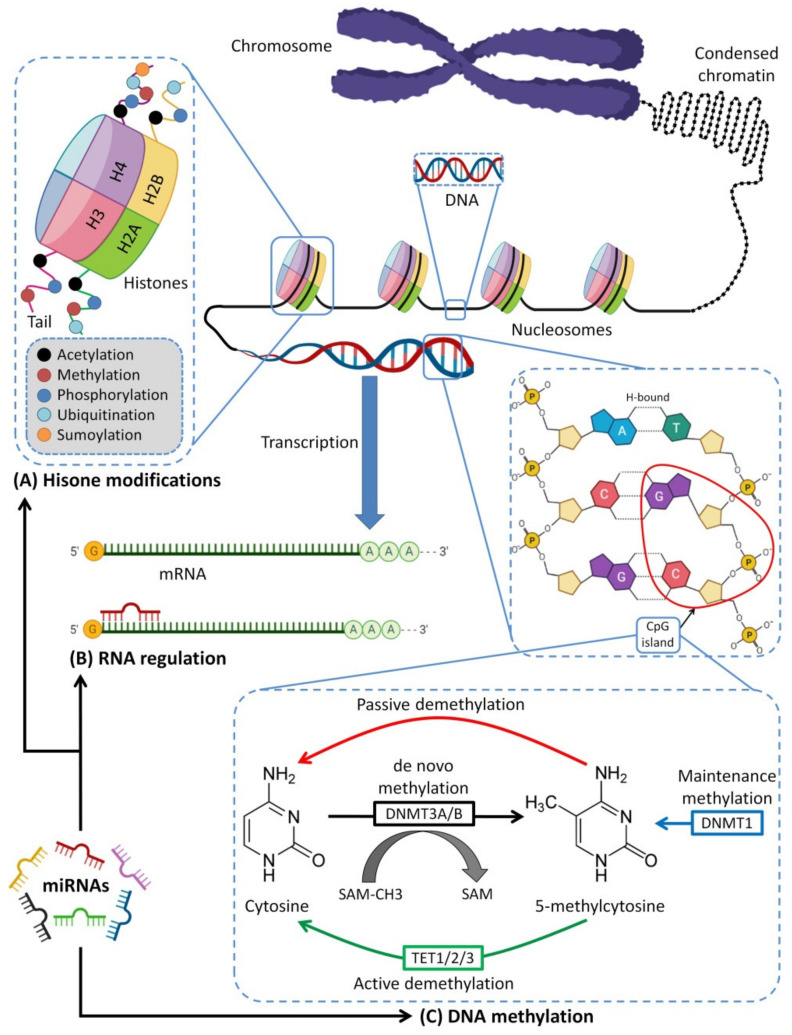
A schematic diagram of the epigenetic mechanisms that can be modulated by miRNAs. Without changing the DNA sequence, miRNAs affect gene expression post-transcriptionally, resulting in altered protein levels of target mRNAs. This effect, known as epigenetic regulation, can occur through three major epigenetic mechanisms, including DNA methylation, histone modifications and RNA regulation. (**A**) Rearrangement of the core histone proteins (H2A, H2B, H3 and H4) involved in chromatin reorganization and regulation of transcription, which come together to form one nucleosome, called histone modifications. These covalent modifications are driven by post-translational addition or removal of acetyl, methyl, phosphate, ubiquityl and sumoyl that attach to the tails of histone proteins. miRNAs could regulate histone modifications by targeting histone-modifying enzymes such as deacetylase and demethylases. (**B**) RNA regulation is a less well-known epigenetic mechanism and occurs through several models, including miRNA regulation of gene expression upon interaction with targeted mRNAs, as shown in Figure 5. Moreover, it has been shown that RNA regulation is involved in epigenetics by modulating chromatin structure. (**C**) The most widely discovered epigenetic mechanism is DNA methylation. It predominantly occurs on cytosine-phosphate-guanine (CpG) dinucleotides called CpG islands (CGIs). DNA methylation patterns start with DNA methyltransferase (DNMTs) enzymes transferring a methyl group from the methyl donor S-adenyl methionine (SAM), which is derived from ATP and methionine, to the fifth carbon of cytosine (on a CGI) to produce 5-methylcytosine (5-mC). DNA methylation patterns involve four processes: (1) adding a methyl group to unmethylated DNA by DNMT3A and DNMT3B (de novo methylation); (2) preserving DNA methylation by DNMT1 during cellular DNA replication (maintenance methylation); (3) inhibition of maintenance methylation (passive demethylation); (4) oxidation and deamination of 5-mC to obtain an unmodified cytosine by the ten–eleven translocation (TET) enzymes 1/2/3. miRNAs can directly target DNMTs (e.g., DNMT3A and DNMT3B) and methyl-CpG binding proteins (e.g., MeCP2 and MBD2), resulting in DNA methylations [674,675,676,677,678,679,680,681]. Created with BioRender.com, accessed on 22 April 2022.

Moreover, Khraiwesh et al. proposed that the initiation of epigenetic silencing by DNA methylation is controlled by the ratio of the miRNA to its target mRNA [670]. In addition to the regulatory effects of miRNAs on DNA methylation, miRNAs can also mediate histone deacetylases (HDACs) modifications. For example, miRNA-1, miRNA-206 and miRNA-140 directly affect HDAC4 regulation. HDAC4 was shown to be a direct target of miR-1 and miR-140 in several research works [671,672]. HDAC4 contains two binding sites for miRNA-1 at its 3′UTR. Another study by Williams et al. [673] showed that amyotrophic lateral sclerosis (ALS) progression disease extensively depends on miRNA-206 level; miR-206 is shown to delay the progression of ALS by targeting HDAC4. The mechanisms of epigenetic regulation are shown in Figure 7.

The initiation of distinct innate and adaptive immune responses can be affected and regulated by epigenetic mechanisms [682]. Epigenetic modifications can affect immune responses by improving the expression of immune-related genes and tumor-associated antigens, besides controlling the secretion of inflammatory mediators such as cytokines and chemokines that participate in the activation of the immune system [683]. Thus, the epigenetic system can produce more prominent immune responses and control the magnitude of inflammation and host immune responses. Moreover, epigenetics systems control other functions in immunity, such as producing monocyte-derived DCs, playing a role in the differentiation of “helper” CD4^+^ T cells and regulatory T cells, generating a memory T cell phenotype and also generating antibodies using plasma cells [682]. Epigenetic modifications, such as the regulation of cytokine production, DNA methylation/demethylation and numerous types of histone modifications in *IFNG*, *IL-4* and *IL-13* genes are involved in the differentiation of Th1 and Th2 cells from naive CD4^+^ T cells. The promoter of *INFG* is hypermethylated in naïve T cells and is only demethylated when the differentiation occurs in Th1 cells, while the promoter of IL4 is hypermethylated in naive T cells and Th1 cells, and only partial demethylation for Th2 cells [684,685].

The epigenetic mechanisms that are supposed to control the memory of the environmental impact may also promote the persistence of disease-associated phenotypes, even in the lack of the initial trigger [682]. There are also various examples of the link between epigenetic changes and autoimmune disorders. DNA hypomethylation of histone deacetylase (HDAC) 1 and 2 levels, hyperacetylation of histones 3 and 4 and decreased methylation rate of histone 3 at lysine 9 (H3K9) have been observed in synovial tissues of patients with rheumatoid arthritis (RA). Additionally, in patients with multiple sclerosis (MS), decreased DNA methylation in the central nervous system white-matter has been detected in comparison to healthy individuals [686]. Moreover, in systemic lupus erythematosus disorder, the prime targets of auto-antibodies are hypomethylated apoptotic DNA and modified histones [687]. Many studies have established a strong association between epigenetics alterations and allergic conditions such as asthma [688]. In paternal origin traits, asthma and epigenetic changes have been found to be passed more from an asthmatic mother than an asthmatic father [689], which can be related to the direct immune interactions between the fetus and its mother [690].

Indeed, various functions of immune cells such as hematopoietic lineage, antigen-receptor rearrangement, exclusion of allelic and immune response induction against pathogens are epigenetically controlled. Furthermore, understanding the epigenetic mechanisms involved in regulating the immune response could be very useful in the explanation of the immunological processes involved in the rejection and allograft tolerance and in developing novel therapeutic approaches [691].

### 7.2. Epigenetic Effects of HBM

In DNA methylation, a methyl group is added to the fifth position of the cytosine ring [10]. In many organisms, DNA methylation is essential for normal organism growth and cellular differentiation. The gene expression pattern in cells is permanently altered by DNA methylation. For example, cells programmed to be liver cells during embryonic development remain as liver cells throughout the organism’s life. DNA methylation is most commonly found in the context of the CpG dinucleotide. In mammals, over 75% of all CpGs are methylated. Over time, methylated C residues spontaneously deaminate to yield T residues. Therefore, CpG dinucleotides slowly mutate to TpG dinucleotides, as indicated by the human genome’s underrepresentation of CpG dinucleotides (only 21% of the expected frequency). Spontaneous deamination of unmethylated C residues, on the other hand, results in U residues, a mutation that the cell rapidly recognizes and corrects [692,693]. MiRNA-mediated post-transcriptional regulation and transcriptional control by epigenetic changes work together to regulate gene expression and sustain physiological functioning. If this circuit is interrupted, it can lead to a variety of diseases [10,173,190].

**Figure 8:**
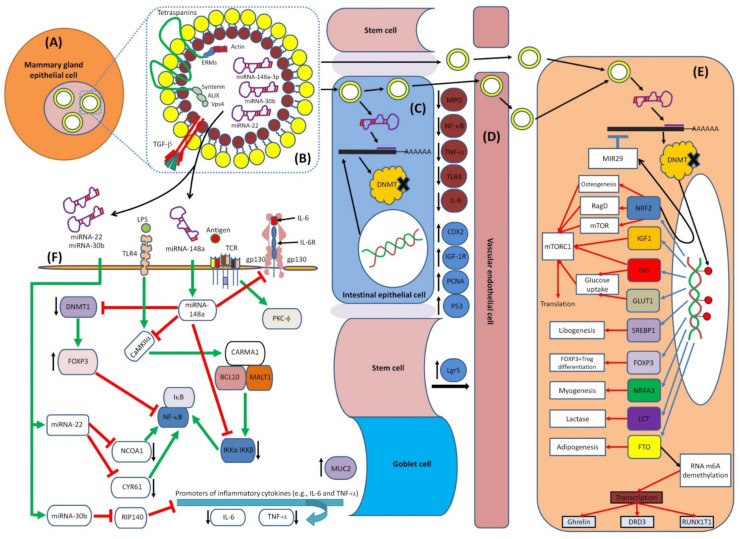
The role of lactation-specific exosomal miRNAs in targeting DNA methyltransferases (DNMTs) in the recipient milk. Exosomes are released by (**A**) mammary gland epithelial cells (MEC) and taken up by a variety of cells, including intestinal epithelial cells (IEC), vascular endothelial cells (VEC), systemic circulation and other body cells [700]. The majority of HBM miRNAs come from MECs, resulting in distinct fractionated milk miRNA profiles [185]. (**B**) The bilayer membrane is critical for MEX resistance to the gastrointestinal tract’s harsh conditions, where miRNA-148a-3p is the main miRNA of MEX. Other important constituents of MEX are transforming growth factor-β (TGF-β) and Tetraspanins such as CD63, CD81, CD9 and CD83 [701,702]. (**C**) HBM exosome (MEX) boosts IEC proliferation, goblet cell proliferation and activity and increases the activity and viability of intestinal stem cells by upregulating the stem cell marker leucine-rich-repeat-containing G-protein coupled receptor 5 (Lgr5) [703]. MEX promotes mucus formation, increases mucin 2 (MUC2) synthesis and decreases nuclear factor κB signaling, tumor necrosis factor-α (TNF-α), toll-like receptor 4 (TLR4), myeloperoxidase (MPO) and interleukin 6 (IL-6) to mediate anti-inflammatory activities. MEX also helps to maintain the antimicrobial barrier by upregulating the antibacterial lectin regenerating islet-derived 3y (RegIIIγ) and inducing the production of tight junction proteins. MEX also interacts directly with bacteria in the gut microbiome [702]. (**D**) Endocytosis by VEC [704] supports the idea that milk-derived exosomes and their miRNA cargo could reach the milk recipient’s systemic circulation and peripheral tissues [700,705,706]. (**E**) Milk exosomes can cross IEC intercellular gaps, which are linked to increased intestinal permeability, especially during the postnatal period. After entering systemic circulation, milk exosomes may reduce DNA methylation of peripheral target cells, where miRNAs induce DNA promoter demethylation of important CpG islands implicated in the activation of gene expression of key transcription factors such as nuclear factor erythroid 2-related factor 2 (NRF2), sterol regulatory element-binding protein-1 (SREBP1), forkhead box P3 (FOXP3) and nuclear receptor subfamily 4 group a member 3 (NR4A3) [707,708]; metabolic regulators such as insulin gene (INS), insulin-like growth factor-1 (IGF1), caveolin 1 (CAV1), glucose transporter 1 (GLUT1) and lactase gene (LCT) [709,710,711,712,713,714]; as well as the RNA m6A demethylase (fat mass- and obesity-associated gene (FTO)), which promotes FTO-dependent mRNA transcription and mRNA splice variant synthesis, such as the adipogenic short version of runt-related transcription factor 1 (RNX1T1), by removing m6A marks on mRNAs. Moreover, Ghrelin and dopamine receptor 3 (DRD3) mRNAs are targeted by FTO-mediated upregulation. The resultant hyperphagia encourages milk consumption to meet newborn growth needs [700,715]. (**F**) Anti-inflammatory actions of miRNA-148a and miRNA-22 and DNMT1 on nuclear factor κB signaling. MiRNA-148a increases the expression of FOXP3, a negative regulator of nuclear factor B, via suppressing DNA methyltransferase 1 (DNMT1). MiRNA-148a targets calcium/calmodulin-dependent protein IIα (CaMKIIα), which phosphorylates CARD-containing MAGUK protein 1 (CARMA1) implicated in IκB kinase α (IKKα) and IκB kinase β (IKKβ) activation. MiRNA-148a, in particular, targets IKKα and IKKβ directly, thereby boosting the inhibitory impact of IκB on NF-κB. Furthermore, miRNA-148a targets the interleukin 6 (IL-6) signal transducer gp130. Nuclear receptor co-activator 1 (NCOA1) and cystein-rich protein 61 (CYR61), which activates NF-kB, are targets of miRNA-22, which is substantially abundant in preterm MEX. IL-6 expression is suppressed by miRNA-30b via targeting RIP140. As a result, miRNAs generated from MEX and DNMT1 inhibition provide anti-inflammatory signaling [701,702,716,717,718].

It was shown that breastfeeding affects DNA methylation of the human genome, especially genes that are involved in the immune response, particularly innate immunity attributed mainly to miRNA-148a-3p, miRNA-146b-5p and others [188]. A main function of 148a-3p is interfering with the function of DNA methyltransferase 3b (DNMT3b), which is important for de novo methylation during the embryonic stage of fetal development and for DNA methyltransferase 1 (DNMT1)-mediated methylation of the DNA after delivery [188,694]. It was found in mice where the knockout of DNMT3b promotes lymphomagenesis due to demethylation of the enhancer gene MENT (also known as Gm128) in normal thymocytes [695]. Changes in DNA methyltransferase (DNMT 1, DNMT 2 and DNMT 3) expression in the liver and skeletal muscle were shown to affect global DNA methylation in the offspring of pigs fed with a low-protein maternal diet [696,697,698,699]. These results may reveal the effect of the maternal diet on carbohydrate and fat metabolism. Figure 8 represents the main immunoregulatory functions of HBM-derived exosomal miRNA and their modulatory effects on DNTMs.

**Figure 9:**
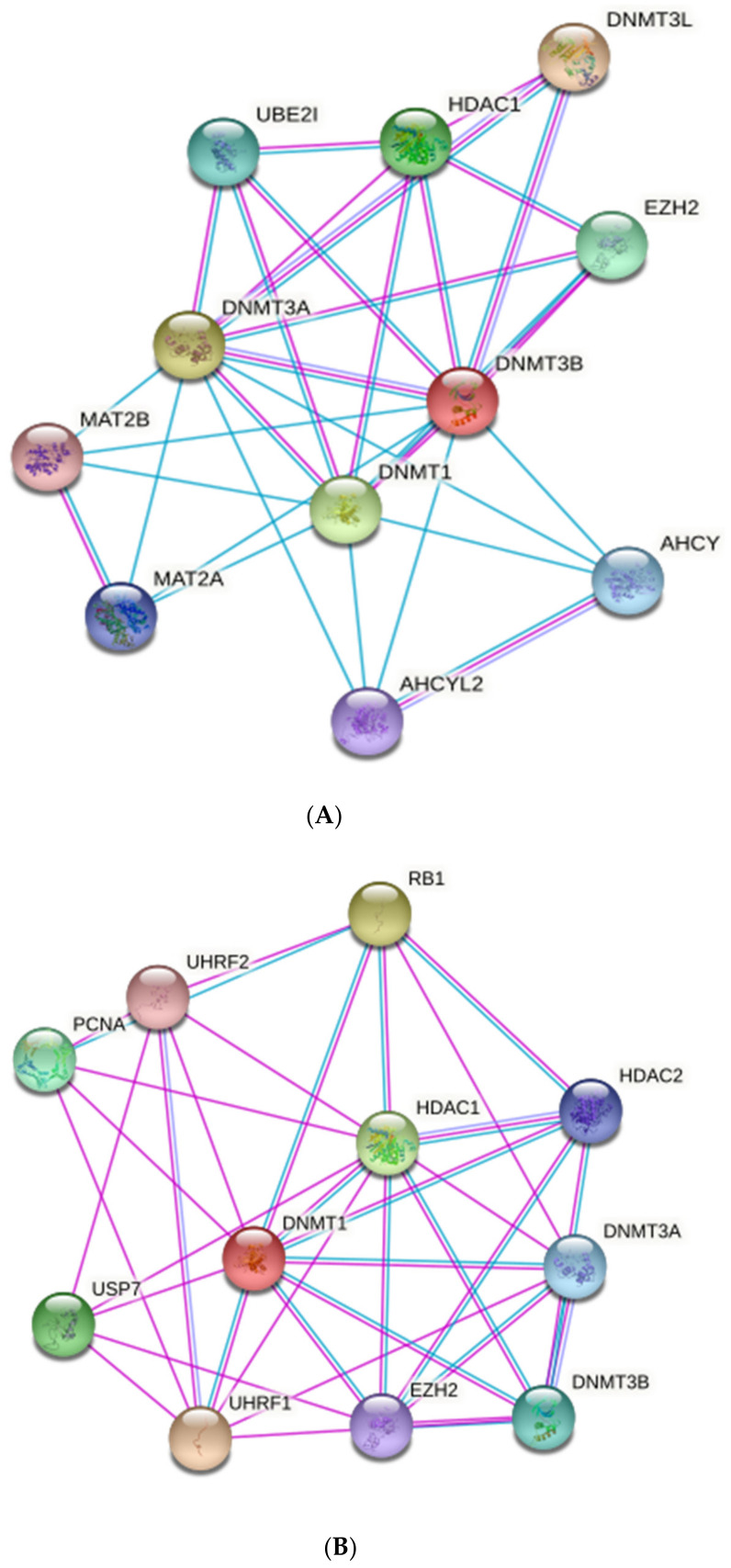
The interaction between DNMT3b (**A**) and DNMT1 (**B**) with other proteins. The edges indicate both functional and physical protein associations. Settings included a minimum interaction score of 0.4. Max number of interactions was 10 in the first shell and 0 in the second shell. Active interaction sources included curated databases and experimentally determined data. Dnmt3L, Dnmt3a and Dnmt3b interact in vitro and in vivo with histone deacetylase HDAC1 [721]. In cancer cells, EZH2 was found to interact with DNMT1, DNMT3A and DNMT3B [722], resulting in hypermethylation of genes, causing more silencing of target genes [723]. However, the exact association of EZH2 with DNMTs remains controversial. Endogenous DNMT1 is sumoylated on many lysine residues (645–1113) in the BAH domains by UBE2I. This improves DNMT1’s catalytic action on genomic DNA [724]. AHCY was discovered as a partner of DNMT1 during the cell cycle of HeLa cells in proteomic analysis. Methyltransferase studies revealed that AHCY increases DNMT1 activity in vitro, while AHCY overexpression in HEK293 cells causes a widespread increase in DNA methylation in vivo [725]. AHCYL2 is homologous to IRBIT and regulates ion-transporting proteins. It is a potential regulator of NBCe1-B in mammalian cells [726]. However, its function remains unclear. The methylation of AHCYL2 gene was shown to be associated with tumors. AHCY, denosylhomocysteinase; AHCYL2, AHCY like 2; MAT, methionine adenosyltransferase; EZH2, enhancer of zeste homolog 2; HDAC1, histone deacetylase 1; HDAC2, histone deacetylase 2; UBE2I, ubiquitin-conjugating enzyme 2I; DNMT, DNA methyltransferase; UHRF1, ubiquitin-like with PHD and ring finger domains 1; USP7, ubiquitin-specific protease 7; PCNA, proliferating cell nuclear antigen; RB1, RB transcriptional corepressor 1. Pink and cyan lines indicate interactions experimentally determined and from curated databases, respectively. Illustrations created using STRING database.

DNMT3b is required for genome-wide de novo methylation and the creation of DNA methylation patterns [719]. DNA methylation is coordinated with histone methylation. It may methylate nucleosomal DNA within the nucleosome core region preferentially, and it may act as a transcriptional co-repressor by interacting with CBX4. It appears to be involved in gene silencing and, in conjunction with DNMT1, to be involved in the stimulation of BAG1 gene expression via the recruitment of CTCFL/BORIS [720]. Figure 9 shows the main interactions of DNMT3b and DNMT1.

It is important to note that studies investigating the effects of breastfeeding are considerably diverse in terms of participant age, tissue investigated, DNA methylation targets and methodologies used for DNA methylation profiling, and breastfeeding categorization [727,728,729,730]. In a study where early exposures were investigated in relation to methylation of cancer-related genes in a case–control study of women with BC [728], it was found that premenopausal women who were never breastfed were nearly three times more likely to have promoter methylation in the *p53* gene (an essential tumor suppressor) [728].

An epigenome-wide association study reported a link between breastfeeding duration and methylation levels at 4276 CpG sites, which are linked to 2635 genes [731]. These genes were primarily involved in the modulation of cell signaling systems, the formation of anatomical structures and cells and, most importantly, the development and function of the immune system and the CNS. These findings led to the conclusion that the oxytocin signaling pathway plays a unique role as a potential activator of coordinated epigenetic modifications in genes involved in CNS function in response to breastfeeding [731]. Breastfeeding appears to influence global methylation patterns, modulate epigenetic effects of some genetic variants and be negatively associated with promoter methylation of the leptin (*LEP*) (an anorexigenic hormone that regulates growth, hunger and insulin sensitivity), *CDKN2A* (implicated in tumor suppression) and Slc2a4 (which encodes an insulin-related glucose transporter) genes and positively associated with promoter methylation of *Nyp* gene (which produces an orexigenic neuropeptide) [732].

For the *LEP* gene, the methylation of its promoter was studied in toddlers in relation to breastfeeding duration [733]. Children who were breastfed for at least 1 to 3 months had lower LEP promoter methylation in white blood cells and higher serum levels of leptin than children who were never breastfed. Methylation of *LEP* was similarly reduced in children with higher birthweights [727]. Both total and exclusive breastfeeding duration were linked to DNA methylation at four *LEP* CpG sites at 10 years old but not at 18 years. Differential methylation region (DMR) analysis found five significant differentially methylated regions in the genome. The duration of breastfeeding was also linked to an early transitory overweight trajectory. *LEP* DNA methylation was also linked to this trajectory at one CpG position, and persistent obesity was linked to another; however, mediation analysis was not significant [733].

Furthermore, the impact of breastfeeding on newborn growth and methylation of obesity-related genes of buccal cells was investigated [734]. There was a significant difference in infant growth and buccal *RXRA* and *LEP* gene methylation after 12 months of breastfeeding. A positive association was identified between *RXRA* CpG2 methylation and breastfeeding duration. For CpG3 and CpG islands of the *RXRA* gene, methylation levels were significantly reduced for children breastfed for 4–6 months compared to non-breastfed ones (only CpG3), and those breastfed for 7–9, 10–12 or 1–3 months. However, children breastfed for 7–9 months (6.1%) had more *LEP* CpG3 methylation than those breastfed for 1–3 months (4.3%) and 10–12 months (4.6%). Furthermore, children who were breastfed for 10–12 months had much lower weight [734].

The relationship between the hypermethylation of *sorting nexin 25* (*SNX25*) gene and allergic disease is exciting [735,736]. Increased promoter methylation causes a decrease in gene expression and, as a result, protein downregulation. *TGF-β* has been demonstrated to be downregulated by *SNX25* [736,737]. *TGF-β* is a regulatory cytokine connected to immune regulation and inflammation that is detected in HBM. TGF-β has been implicated in the pathophysiology of allergic diseases [736,738,739].

Two differentially methylated positions (DMPs) in the genes *SNX25* and *LINC00840* were shown to be substantially linked with breastfeeding duration of more than 6 months, and the findings were reproduced for exclusive breastfeeding for more than 3 months. Furthermore, in 10-year-old children who were breastfed for more than three months, a substantial differentially methylated region (DMR) covering the *FDFT1* gene was discovered [735]. During infancy, exclusive breastfeeding causes more significant DNA methylation changes than during later stages of child development. At the genome-wide analysis, 2 CpG sites in boys (*NREP* and *IL16*) and 13 CpG sites in girls (*ATP6V0A1*, *DHX15/PPARGC1A*, *LINC00398/ALOX5AP*, *FAM238C*, *miR-21*, *SNAPC3*, *NATP/NAT2*, *CUX1*, *TRAPPC9*, *OSBPL1A*, *ZNF185*, *FAM84A*, *PDPK1*) were found to mediate the association between longitudinal BMI and exclusive breastfeeding. Enrichment of CpG sites located within miRNAs and key pathways (*AMPK* signaling pathway, insulin signaling pathway and endocytosis) was detected. In a dose–response relationship with exclusive breastfeeding time, total DNA methylation variation corresponding to 3 to 5 months of exclusive breastfeeding was related to lower BMI growth in the first 6 years of life [740].

The number of weeks of breastfeeding in young women in the United Kingdom had minimal, non-statistically significant effects on methylation of the interleukin-4 receptor gene’s relevant CpG island, which was associated with asthma risk [730]. Various degrees of breastfeeding (none, less than 3 months, more than 3 months) were connected to different patterns of whole-genome methylation in a case–control study of asthma in 200 children from industrial vs. rural settings in the Czech Republic [729]. Another study looked at the age-related rise in global methylation in blood at birth, 7 and 17 years, in relation to a range of maternal, pregnancy and birth-related variables, such as whether the child had ever been breastfed. In this study, there was no significant association between breastfeeding and methylation differences [741].

Breastfeeding may potentially expose infants to epigenetic consequences from the mother’s environment or health habits. When compared to individuals who did not breastfeed, the methylation of *DRD4* (a key dopamine receptor) in cheek cells was higher in eight-week-old children whose moms drank moderate amounts of alcohol during breastfeeding compared to those who did not drink [742]. Despite the fact that the brain is the most important tissue for studying dopamine-receptor methylation, sampling live infants is clearly invasive. Relapsed adult alcoholics exhibit equivalent alterations in the methylation of cheek-cell dopamine receptors because cheek cells are formed from the same primordial germ layer as the brain [743].

Separate linear regression models controlling for confounders were used to identify 87 differentially methylated CpGs in different breastfeeding and formula feeding children (exclusive breastfeeding (EBF): 27 CpGs, exclusive formula feeding (EFF): 48 CpGs and mixed: 12 CpGs) [744]. The EFF group had a significantly lower total of all methylation alterations from birth to the age of ten years old. As a result, the number of CpGs with a methylation reduction increased by 4.7% (13,683 CpGs). Future research is needed to lessen the negative health impacts of lower methylation associated with exclusive formula feeding and its negative potential for a child’s development [744]. Breastfeeding is linked to epigenetic changes in buccal cells in children. After controlling for child and maternal factors, four significant CpGs were related to breastfeeding in the subgroup of children less than ten years [745]. Methylation differences at these CpGs were smaller and non-significant in children beyond the age of ten years. Three of the previously published CpG sites were linked to breastfeeding in children under the age of ten years, indicating that these CpGs are linked to breastfeeding in buccal and blood cells [745].

Furthermore, researchers looked into the associations between breastfeeding length and DNA methylation at two sites in the promoter of the toll-like receptor-1 (*TLR1*) gene, as well as the link between *TLR1* DNA methylation and illness risk [746]. Blood was drawn from 100 adults and divided into two groups based on the length of time they were breastfed (<6 months and ≥6 months), with 53 samples undergoing DNA extraction. This study found a significant association between longer breastfeeding length and decreased susceptibility to influenza and allergies, as well as a significant reduction in DNA methylation within the TLR1 gene promoter [746].

Researchers reported two differentially methylated sites to have directionally consistent associations with breastfeeding at the ages of 7 and 15–17 years, but not at birth [747]. Twelve differentially methylated regions were found in relation to breastfeeding, three of which showed signs of directional concordance with ages 7 and 15–17 years, but not at birth and age 7 years [747]. A study investigated whether DNA methylation, which is influenced by dietary intake, could play a role in the link between breastfeeding and child cognition [748]. The goal was to see if there was an association between global DNA methylation and cognition and behavior in 4-year-old children (73 children). No association was found between global DNA methylation and child behavior and cognition [748].

The impact of dietary intervention in the first year of life on global methylation levels in leukocyte samples from a group of infants born in southern Brazil between 2001 and 2002 was investigated [749]. Overall methylation assessments were performed on DNA samples from 237 4-year-old children using enzyme-linked immunosorbent assays. The intervention group’s mean methylation values were greater compared to the control group. As a result, these factors may play a role in the rise in global DNA methylation. The findings of this study support the idea that nutritional interventions should be made early in life [749]. In another study, Anderson et al. [750] found that maternal vitamin D supplementation affects the methylation of genes involved in developmental processes in mothers and breastfed babies. Loss of methylation was linked to genes involved in metabolic functions and signal transduction pathways. [750]. Table 4 summarizes studies that investigated the epigenetic effects of breastfeeding on different physiological and pathological functions.

Munch et al. [189] found that the genetic targets of 308 miRNAs in HBM had different functions, including gene expression and metabolic control, as well as immunological responses, implying that these miRNAs may be important for HBM-fed infants [189,752]. In addition to these roles, several miRNAs are thought to play a role in central nervous system (CNS) regulation. It has also been observed that HBM miR-118.2 targets Teneurin Transmembrane Protein 2 (TENM2), which is found in elevated concentrations in the CNS [753], implying a regulatory role in the infant’s brain nervous system connection development [754,755]. MiRNAs generated from milk can also be used to target adipogenesis in infants. Overexpression of miRNA-155 may target the adipogenic transcription factor CCAAT/enhancer-binding protein-β (C/EBPβ), which results in reducing the mass of brown adipose tissue [756]. Furthermore, milk-derived miRNA-29a is able to inhibit the *INSIG-1* gene [189,190,757], which regulates adipogenesis processes [758]; this has been linked to body mass index (BMI) [759]. In addition, milk miRNAs also play a significant role in the control of short- and long-term appetite of breastfed infants via numerous appetite regulatory hormones of HBM, such as adiponectin, leptin, insulin, ghrelin and others [760].

HBM-derived miRNAs have been hypothesized to protect children from cancer from birth to adulthood [189]. MiR-21, for example, is found in both human and bovine milk [188] and has been shown to be overexpressed in human hepatocellular carcinoma (HCC). Any dysregulation of miR-21 can be linked to HCC increase by altering mTORC1 signaling, such as PTEN expression [761]. MiR-21 is a common miRNA found in bovine milk [757], as well as mature and colostrum HBM [190]. It is also plentiful in human plasma [762] and is assumed to have a role in postnatal growth promotion in newborns [705]. MiR-21 also has additional normal tissue activities, such as regulating adipogenic development in human adipose tissue mesenchymal stem cells (MSCs) [763]. Furthermore, HBM miRNAs may influence tumor suppressor genes directly [764], such as the let-7 family, which inhibits the growth of lung tumors by targeting the RAS oncogenes directly [765]. Further research into the natural roles of miRNA in the infant and the lactating breast is needed. Furthermore, based on the target predictions of novel-miR-118 in the nervous system, it seems that the presence of novel-miR-118.2 in HBM has neurocognitive advantages [189].

## 8. Conclusions

HBM contains several physiologically active components that aid in newborn growth, development and health. Most research studies have focused on milk exosomes and their miRNA content. The miRNAs in HBM emerged as potential immune-regulatory agents targeting the immune cells, influencing the development of an infant’s immune system through immune modulation. Moreover, miRNAs play crucial roles in modulating the expression of a wide range of genes by targeting DNA methyltransferases. Physiological miRNA transfer during breastfeeding delivers suitable signals for sufficient epigenetic programming of newborns.

## Figures and Tables

**Table 1:** The main immunomodulators in HBM and their roles in improving the health and immune system.

Component	Types	Major Immune-Related Functions	Reference
Fatty acids	- Monounsaturated (42%) - Medium-chain (42%) - Long-chain polyunsaturated (16%); linoleic acid (10%), arachidonic acid (1%), α-linolenic acid (>1%), eicosapentaenoic acid (0.1%), docosahexaenoic acid (0.4%), and others (3.7%)	- Maturation of immune system - Modulate the acquired immunological responses that affect the balance between Th1 and Th2 cells and Treg and Teff cells - Regulate the production of immunomodulatory cytokines (e.g., TGFs) - Enhance level of innate immune response (i.e., soluble CD14) and adaptative immune response (i.e., IgA) - Act as antiviral, antibacterial and antiprotozoal agents	[98,99,100,101]
Oligosaccharides	- Fucosylated (35% to 50%) - Sialylated (12–14%) - Nonfucosylated neutral (42–55%)	- Influence the expression of chemokines (e.g., CX3CL1, CCL5, CXCL2, CXCL3), cytokines (e.g., IL-4, IL-17C, IL-8, IL-1β, IL-10, IFN-γ), cellular receptors (IFNGR1), cell adhesion molecules (e.g., ICAM-1/2) - Reduce the infectivity of rotavirus, norovirus and influenza viruses - Exhibit antimicrobial and antibiofilm activities against *S. agalactiae, E. coli, B. subtilis and S. aureus* - Shape the gut microbiota in infants - Inhibit leukocyte adhesion to endothelial cells - Modulate TLR-4 signaling - Induce the production of cytokines (i.e., IL-4 and IFN-γ) required for expansion of Th1 and Th17 implicated in the pathogenesis of enterocolitis - Increase serum levels of IgG1 and IgG - Increase the expression of CD27 on splenic B-cells - Inhibit the adherence and binding of specific pathogens to the host cells in intestine	[19,102,103,104,105,106,107,108,109]
Hormones	Leptin, erythropoietin, adiponectin, ghrelin, IGFs, resistin and obestatin	- Erythropoietin prevents HIV transmission from mother to child - Adiponectin reduces inflammation, regulates infant metabolism (reducing later-life obesity) and inhibits production of TNF-α in intestinal epithelium and macrophages - Leptin regulates proinflammatory cytokines (e.g., TNF-α and IL-6) and Th1 responses; it also promotes proliferation and activation of monocytes and NK cells, neutrophils chemotaxis and T cell survival by modulating the expression of anti-apoptotic proteins (e.g., Bcl-xL) - IGFs play vital roles in the development and function of T cells - Resistin is involved in the anti-infection immune process by interacting with a variety of immune cells; can either directly or indirectly promote infiltration, adhesion and migration of monocytes, neutrophils and CD4^+^ T cells	[19,35,110,111,112,113]
Cells	Leukocytes (i.e., lymphocytes, neutrophils and macrophages), hematopoietic stem cells and hematopoietic progenitor cells	- Maternal leukocytes provide active immunity by fighting pathogens via phagocytosis and intracellular killing, produce microbicidal molecules, present antigens; also play vital role in shaping infant’s immune system, promoting development of immunocompetence and altering gut bacterial colonization	[19,114,115]
Proteins, glycoproteins and peptides	Cytokines, chemokines, soluble receptors, receptor agonists and antagonists, growth factors, immunoglobulin and others	- They enhance defense against pathogenic bacteria, viruses and yeasts and promote gut development and immune function. Cytokines and chemokines are the most redundant secreted proteins that provide active immunity to infants. For example, TGF-β prevents diseases induced by allergy and controls wound repair and inflammation; G-CSF plays a role in sepsis treatment and enhances cell prefoliation, crypt depth and villi; IL-6 (a key circulating pyrogen) activates CNS mechanisms in fever during infection and inflammation; IL-7 helps develop thymic; IL-8 protects from TNF-α-induced damage; IL-10 has anti-inflammatory activity; IFN-γ has pro-inflammatory activity as it inhibits the Th2/allergic response while increases the Th1/inflammation response - HBM contains glycoprotein cytokine receptors that modulate immune responses. For example, sTNFR1/2 and IL1Ra suppress pro-inflammatory TNF-α and IL-1 activity, respectively, decrease stimulation of IL-8 secretion from the intestinal epithelial of neonates and reduce necrotizing enterocolitis; TLR1-9 agonist and antagonist protect the infant from infections - Growth factors: TGF-β induces regulatory T cell production; epidermal GF inhibits apoptosis in intestinal cells; NGF enhances the outgrowth and survival of neurons; IGF alters intestinal atrophy, induces erythropoiesis; VEGF plays a role in angiogenesis regulation, decreasing the burden of premature retinopathy - Immunoglobulins: IgG, IgM and IgA account for 90% of HBM immunoglobulins and provide passive immunity to the newborn. The major function of IgA and IgG is neutralization of pathogens by binding to them and preventing them from binding to the epithelial cells in the gut mucosa. Moreover, by opsonizing the antigen for complement fixation and destruction, IgM suppresses microbial infections - Bile salt-dependent lipase blocks viral infection (such as HIV) by binding to the pathogen receptor DC-SIGN - Mucin1/4 protect infant from viral (e.g., rotavirus, norovirus and HIV) and bacterial (e.g., *E. coli* and *S. enterica*) infections - Cathelicidin-derived antimicrobial peptides produced by cells in breast milk protect infant from autoimmune diseases and have broad antimicrobial activities against Gram-positive and Gram-negative bacteria - α-lactalbumin is the major protein found in HBM that is converted in the stomach to HAMLET. In the presence of free oleic acid, HAMLET reduces the volume of >95% of skin papilloma - Soluble CD14 sensitizes the innate mucosal immune system to Gram-negative bacteria, such as *E. coli,* and mediates TLR4 binding to lipopolysaccharide of Gram-negative bacteria; inhibits TLR2 signaling and attenuates TLR4 signaling - HβD-2 is a peptide that inhibits TLR7 signaling and has antibacterial activities against *Salmonella* spp., *E. coli* and *P. aeruginosa* - Lactoferrin is a glycoprotein that has capacity against various fungi, viruses and bacteria; inhibitory effects reported against *V. cholera* and *E. coli*; responsible for sequestering iron needed by bacteria for growth and survival; influences TLR4 signaling - Lactadherin is a glycoprotein that protects neonates from rotavirus infection, mediates phagocytosis of apoptotic cells, blocks NF-κB and TLR4 signaling leading to a signaling cascade that reduces inflammation	[19,98,110,116,117,118,119,120,121,122,123]
Lysozymes		- Lysozymes hinder growth of many bacterial species by disrupting the proteoglycan layer of the cell wall - Lysozymes are characterized by a positive charge which can facilitate electrostatic interactions with the viral capsid blocking the viral fusion proteins (especially in HSV and HIV)	[124,125,126]
Nucleotides	CMP, UMP, GMP, AMP	- Enhance immune responses and promote the development of a less pathogenic intestinal flora in infant	[127,128]
Nucleic acids	- DNA fragments - ncRNAs, including miRNA, siRNA, lncRNA, circRNA, piRNA, rRNA and tRNA	- miRNAs have direct impacts on immunological regulation, such as suppressing the production of essential transcription factors in immune cell polarization or altering the epigenetic state of immune cell lineages - Other ncRNAs are less studied than miRNA but have been found to be functionally involved in several regulatory mechanisms related to miRNA mechanisms and mRNA translation process	[129,130]

Abbreviations: DC-SIGN, dendritic cell-specific intercellular adhesion molecule-3-grabbing non-integrin; HAMLET, human α-lactalbumin made lethal to tumor cells; IGF, insulin-like growth factor; TGF, transforming growth factor; TNF, tumor necrosis factor; Th; T helper cells; Teff, effector T cell; Treg, regulatory T cell; TLR, Toll-like receptor; HBM, human breast milk; HSV, herpes simplex virus; HIV, human immunodeficiency virus; HβD-2, human β-defensin 2; ncRNAs, non-coding RNAs; miRNA, microRNA; siRNA, small interfering RNA; lncRNA, long noncoding RNA; circRNA, circular RNA; piRNA, Piwi-interacting RNA; rRNA, ribosomal RNA; tRNA, transfer RNA; CMP, cytidine monophosphate; UMP, uridine monophosphate; GNP, guanosine monophosphate; AMP, adenosine monophosphate.

**Table 2:** The odds ratio of different diseases among breastfed people compared with commercial infant formula-fed or referent group specified.

Condition	Breastfeeding (Months)	Comments *	OR **
Otitis media	Any	-	0.77
	≥3	Exclusive BF	0.50
Upper RTI	>6	Exclusive BF	0.30
Lower RTI	≥4	Exclusive BF	0.28
Asthma	≥3	Atopic family history	0.60
		No atopic family history	0.74
RSV bronchiolitis	>4	-	0.26
NEC	NICU stay	Preterm infants with exclusive HBM	0.23
Atopic dermatitis	>3	Exclusive BF negative family history	0.84
		Exclusive BF positive family history	0.58
Gastroenteritis	Any	-	0.36
IBD	Any	-	0.69
Obesity	Any	-	0.76
Celiac disease	>2	Gluten exposure when BF	0.48
T2D	>3	Exclusive BF	0.71
	Any	-	0.61
ALL	>6	-	0.80
		-	0.85
SIDS	Any	-	0.64

Abbreviations: ALL, acute lymphocytic leukemia; BF, breastfeeding; HBM, human breast milk; IBD, inflammatory bowel disease; RSV, respiratory syncytial virus; T2D, type 2 diabetes; RTI, respiratory tract infection; NEC, necrotizing enterocolitis; NICU, neonatal intensive care unit; SIDS sudden infants death syndrome. * Referent group is exclusive BF ≥ 6 months. ** OR, odds ratio: expressed as increased risk relative to commercial formula feeding.

**Table 3:** The abundantly expressed miRNAs in HBM and their physiological functions in normal and pathological conditions.

miRNA [Sequence]	Function [Reference]
Colostrum-specific miRNAs	
hsa-let-7i-5p [UGAGGUAGUAGUUUGUGCUGUU]	Regulates cell morphology and migration through distinct signaling pathways in normal and pathogenic urethral fibroblasts [237]; protects against acute ischemic stroke [238]; controls the migration of head and neck cancer cells through downregulation of BMI1 protein [239]; inactivates localized scleroderma [240]; regulates MS pathogenesis by suppressing induction T_reg_ by targeting IGF1R and TGFβR1 [241]; protects against pneumoconiosis caused by nanoparticles inhalation [242]; acts as an autophagy suppressor by targeting ATG10 and ATG16L1 in NPC and may represent a promising therapeutic target for NPC treatment [243]; targets *HABP4* gene and functions as a tumor promoter in ccRCC, and thus offers a potential target for treatment [244]; inhibits granulosa-luteal cell proliferation and oestradiol biosynthesis by directly targeting IMP2 [245]; inhibits KGN proliferation and decreases estradiol production in an IMP2-dependent manner, providing insights into the pathogenesis of PCOS [246]; promotes differentiation of hESCs [247]; inhibits the metastasis of TNBC [248].
hsa-miR-423-5p [AAAAGCUGGGUUGAGAGGGCAA]	Regulates ovarian response to ovulation [249]; targets ING-4 and upregulates signaling molecules such as p-AKT and p-ERK1/2, which support miR-423-5p functions as an oncogene in glioma and suggests targeting it as therapeutic potential for glioma [250]; targets PTTG1 and SYT1 mRNAs, thus induces cell apoptosis, inhibits cell proliferation and reduces growth hormone release and migration of GH3 cells [251]; regulates TGF-β signaling by targeting SMAD2, thus functions in the development of bicuspid aortic valve BAV disease and its complication, bicuspid aortopathy [252]; induces silencing of the nerve growth factor, which promotes retinal microvascular dysfunction, demonstrating the potential for miRNA-based therapy for treating diabetic retinopathy [253]; promotes BC invasion [254].
hsa-miR-320b [UUCAAGUAAUUCAGGAUAGGU]	Negatively regulates normal human epidermal keratinocyte proliferation by targeting AKT3 to regulate the STAT3 and SAPK/JNK pathways, thus might participate in the pathogenesis of psoriasis, may act as a novel diagnostic marker or therapeutic target for this disease [255]; affects HCC radiosensitivity to ionizing radiation treatment through DNA damage repair signaling [256]; regulates osteoblast differentiation [257]; modulates cholesterol efflux and atherosclerosis [258].
hsa-miR-26b-5p [UUCAAGUAAUUCAGGAUAGGU]	Controls the adipogenic differentiation of hADMSC [259]; acts as a tumor suppressor in PC [260]; affects cytokine secretion in RA [261]; modulates Th17 cell plasticity in RA [262]; inhibits proliferation, migration, invasion and apoptosis induction of osteosarcoma cells [263].
hsa-miR-146a-5p [UGAGAACUGAAUUCCAUGGGUU]	Modulates androgen-independent PC cell apoptosis [264]; regulates KIR expression [265]; acts as a tumor suppressor in B-cell malignancies [266]; inhibits the metastasis of ccRCC [267]; inhibits NSCLC proliferation [268]; protects cardiomyocytes and myocardial tissues in polymicrobial sepsis [269]; associated with low-risk human PSCCs [270]; improves the decidual cytokine microenvironment [271]; acts as tumor suppressor in esophageal, prostatic, glioma and ovarian cancers [272,273,274,275]; suppresses osteoclastogenesis [276].
hsa-let-7c-5p [UGAGGUAGUAGGUUGUAUGGUU]	Targets TGF-β signaling and contributes to the pathogenesis of renal fibrosis [277]; inhibits osteo/odontogenic differentiation of IGF-1-treated DPMSCs by targeting IGF-1 [278]; inhibits MAP4K4 expression; inhibits OSCC cell proliferation and migration [279].
hsa-miR-200b-3p [UAAUACUGCCUGGUAAUGAUGA]	Inhibits epithelial-to-mesenchymal transition in TNBC [280]; inhibits human cytomegalovirus replication [281]; acts as a tumor suppressor in HCC [282]; regulates self-renewing divisions in PC cells by inducing less Notch signaling and promotes daughter cells to become asymmetric [283]; promotes endothelial cell apoptosis by targeting HDAC4 in atherosclerosis [284]; inhibits cell proliferation and Ca^2+^ influx in PASMCs [285].
hsa-miR-151b [UCGAGGAGCUCACAGUCU]	Controls expression of GHR [189] and regulates proliferation and apoptosis of THCA cells through SNRPB axis [286].
hsa-miR-24-3p [UGGCUCAGUUCAGCAGGAACAG]	Enhances NPC radiosensitivity by targeting both the 3’UTR and 5’UTR of Jab1/CSN5 [287]; enhances cell growth in HCC by targeting metallothionein 1M [288]; regulates lung adenocarcinoma progression through FGFR3 signaling [289]; regulates neuronal differentiation by regulating hippocalcin expression [290]; inhibits progression of pancreatic ductal adenocarcinoma through LAMB3 downregulation [291]; regulates proliferation, migration and invasion of cancer cells by directly targeting p130Cas [292]; suppresses proliferation and invasiveness of gastric mucosal lesions [293].
hsa-miR-107 [AGCAGCAUUGUACAGGGCUAUCA]	Regulates cellular migration by inducing CDK5 activity and the associated molecular pathways [294]; inhibits acute aortic dissection progression [295]; regulates chemo-drug sensitivity in BC cell by targeting TRIAP1 [296]; downregulates Cdc42 3’UTR and suppresses ESCC proliferation, migration and invasion [297]; modulates *NeuroD1* and *SOX6* genes affecting MSCs commitment toward insulin-producing cells [298]; modulates chondrocyte proliferation [299]; inhibits glioma cell migration and invasion [300]; regulates cisplatin chemosensitivity in NSLCC [301]; promotes tumor suppressor in GC [302]; inhibits endothelial progenitor cell differentiation [303]; contributes to post-stroke angiogenesis [304]; antagonizes profibrotic phenotypes of pericytes [305].
hsa-miR-221-3p [AGCUACAUUGUCUGCUGGGUUUC]	Regulates apoptosis in ovarian granulosa cells [306]; regulates epithelial ovarian cancer progression [307]; affects proliferation and apoptosis of keratinocytes [308]; reduces airway eosinophilia and CXCL17 expression in asthma [309]; targets CACNA1C and KCNJ5 and alters cardiac ion channel expression [310]; downregulates EIF5A2 and inhibits cell proliferation in medulloblastoma [311], acts as a tumor suppressor and disease progression marker in prostate cancer [312]; down-modulates KIT receptor, which suggests a potential role in cancer therapy [313]; regulates CDKN1C/p57 and CDKN1B/p27 expression in HCC [314], suppresses HDAC6 providing a new target for the treatment of liver malignancies [315], targets KIT and ETV1 in gastrointestinal stromal tumors [316].
hsa-miR-151a-5p [UCGAGGAGCUCACAGUCUAGU]	Regulates E-cadherin in NSCLC cells, which promotes partial EMT and thus acts as a therapeutic target [317].
hsa-miR-378c [ACUGGACUUGGAGUCAGAAGAGUGG]	Suppresses stomach adenocarcinoma cell proliferation, migration, invasion and epithelial-mesenchymal transition [318].
Mature milk-specific miRNAs	
hsa-miR-375 [CCCCGCGACGAGCCCCUCGCACAAACCGGACCUGAGCGUUUUGUUCGUUCGGCUCGCGUGAGGC]	Induces generation of insulin-producing cells from human decidua basalis-derived stromal cells [319]; promotes pancreatic cell differentiation [320]; suppresses ESCC by direct targeting of SHOX2 [321], reduces viability of HCC under hypoxic conditions [322], suppresses bladder cancer via the Wnt/beta-catenin pathway [323]; enhances generation of insulin-producing cells from human MSCs [324]; promotes redifferentiation of adult human β cells [325]; enhances infant growth and development [189]; regulates expression of *JAK2* [189]; activates p21; suppresses telomerase activity [326].
hsa-miR-193b-3p [AACUGGCCCUCAAAGUCCCGCU]	Regulates matrix metalloproteinase in chondrocytes [327]; regulates chondrogenesis [328] and chondrocyte metabolism [329]; acts as tumor suppressor in ovarian carcinoma cells [330]; attenuates neuroinflammation in early brain injury after subarachnoid hemorrhage [331].
hsa-miR-345-5p [GCUGACUCCUAGUCCAGGGCUC]	Acts as anti-osteogenic factor [332].
hsa-miR-423-3p [AGCUCGGUCUGAGGCCCCUCAGU]	Activates oncogenic autophagy in GC [333] and enhances tumor growth in lung adenocarcinoma [334].
hsa-miR-125a-5p [UCCCUGAGACCCUUUAACCUGUGA]	Decreases sensitivity of T_reg_ cells toward IL-6-mediated conversion [332]; suppresses breast cancer by downregulating BAP1 [335,336]; suppresses bladder cancer by targeting FUT4 [97]; suppresses cervical carcinoma [337], HCC [338], GC [339] colon cancer [340], prostate carcinoma [341], bladder cancer [336] and CRC [342]; activates p53 and induces apoptosis in lung cancer cells [343,344]; contributes to hepatic stellate cell activation [345]; inhibits trophoblast cell migration and proliferation in preeclampsia [346].
hsa-miR-148a-5p [AAAGUUCUGAGACACUCCGACU]	Regulates expression of SOCS-7 [189]; controls ATPase expression [189]; regulates triacylglycerol and long-chain acyl-CoA fatty acid synthesis [189]; regulates lactose synthesis [189]; promotes cartilage production [347]; relieves hepatic fibrosis [348]; regulates the stem cell-like side population distribution in ESCC [349].
hsa-miR-29c-3p [UAGCACCAUUUGAAAUCGGUUA]	Regulates biological function of CRC [350]; suppresses gallbladder carcinoma [351], T-cell acute lymphoblastic leukemia [352], ovarian cancer [353] and melanoma [354].
hsa-miR-27a-3p [UUCACAGUGGCUAAGUUCCGC]	Regulates expression of intercellular junctions at the brain endothelium and controls the endothelial barrier permeability [355]; suppresses osteoblastogenesis [356]; suppresses OSCCs [357] and HCC [358]; inhibits cell proliferation and inflammation of RA in synovial fibroblasts [356,359]; mediates of human adipogenesis [360].
hsa-miR-365a-3p [UAAUGCCCCUAAAAAUCCUUAU]	Suppresses progression of PC [361] and GC [362].
hsa-miR-365b-3p [UAAUGCCCCUAAAAAUCCUUAU]	Promotes HCC cell migration and invasion [363].
hsa-miR-183-5p [UAUGGCACUGGUAGAAUUCACU]	Modulates cell adhesion [364]; regulates uterine receptivity and enhances embryo implantation [365]; promotes invasion of endometrial stromal cells [366]; regulates myogenic differentiation [367].
hsa-miR-148b-3p [UCAGUGCAUCACAGAACUUUGU]	Stimulates osteogenesis [368] and suppresses glioma cells [369].
hsa-miR-28-3p [CACUAGAUUGUGAGCUCCUGGA]	Inhibits diffuse large B-Cell lymphoma cell proliferation [370].
Common miRNAs	
hsa-miR-141-3p [UAACACUGUCUGGUAAAGAUGG]	Suppresses ameloblastoma cell migration [371]; suppresses osteosarcoma cells [372], GC [373] and CRC [374]; promotes endothelial cell angiogenesis [375]; regulates myogenic differentiation in myoblasts [376]; regulates IL-13-induced airway mucus production [377]; regulates mesenchymal stem cell aging [378].
hsa-miR-22-3p [AAGCUGCCAGUUGAAGAACUGU]	Suppresses endothelial progenitor cell proliferation and migration in venous thrombosis [379]; suppresses T-cells in ALL [380]; suppresses sepsis-induced acute kidney injury [381].
hsa-miR-181a-5p [AACAUUCAACGCUGUCGGUGAGU]	Reduces oxidation resistance in osteoarthritis [382]; suppresses prostate cancer [383]; regulates several cancer genes [384]; regulates multiple malignant processes of breast cancer [385]; suppresses invasion and migration of HTR-8/SVneo in pre-eclampsia [386].
hsa-miR-26a-5p [UUCAAGUAAUCCAGGAUAGGCU]	Regulates the glutamate transporter in multiple sclerosis [387]; regulates fatty acid and sterol metabolism in nonalcoholic fatty liver disease; regulates the expression of inducible nitric oxide synthase in human osteoarthritis chondrocytes [388]; suppresses breast cancer [389] and prostate cancer [390].
hsa-miR-30a-5p [UGUAAACAUCCUCGACUGGAAG]	Suppresses CRC [391], lung squamous cell carcinoma [392], and renal cell carcinoma [393].
hsa-let-7a-5p [UGAGGUAGUAGGUUGUAUAGUU]	Decreases cell proliferation and inhibits the expression of Bcl-2 in ovarian cancer cells [394].
hsa-miR-148a-3p [UCAGUGCACUACAGAACUUUGU]	Suppresses GC [395]; promotes ADH4 expression [396]; regulates angiogenesis [397].
hsa-miR-27b-3p [UUCACAGUGGCUAAGUUCUGC]	Suppresses glioma [398], lung cancer [399], CRC [400]; endothelial cell proliferation and migration in Kawasaki Disease [401]; suppresses Osteogenic differentiation of maxillary sinus membrane stem cells by targeting Sp7 [402].
hsa-miR-146b-5p [UGAGAACUGAAUUCCAUAGGCUG]	Suppresses NSCLC [403], and glioma [404]; down-regulates BRCA1 expression in TNBC [405]; induces IL-6 [406].
hsa-let-7f-5p [UGAGGUAGUAGAUUGUAUAGUU]	Promotes bone marrow MSCs survival in AD [407] and suppresses NSCLC [408].
hsa-miR-21-5p [UAGCUUAUCAGACUGAUGUUGA]	Suppresses breast cancer cells [409]; induces angiogenesis [410]; regulates mesothelin expression [411]; promotes ThP-1 cell proliferation [412]; links EMT in keloid keratinocytes [413].
hsa-miR-92a-3p [UAUUGCACUUGUCCCGGCCUGU]	Suppresses lymphoma [414]; promotes cell proliferation, invasion and metastasis, inhibiting cell apoptosis and serving as predictive biomarkers for tumor diagnosis or chemoresistance [415]; regulates angiogenesis in stromal cells [416]; relates to activated partial thromboplastin time, prothrombin activity and plasma lipocalin-2 level [417]; regulates cartilage-specific gene expression in chondrogenesis [418]; regulates aggrecanase-1 and 2 expressions in human articular chondrocytes [419].
hsa-miR-16-5p [UAGCAGCACGUAAAUAUUGGCG]	Suppresses CRC [420], chordoma [421], neuroblastoma [422] and breast cancer [423]; involved in dilation of ischemic cardiomyopathy [424]; enhances radiosensitivity in prostate cancer [425]; prevents amyloid β-induce injury [426]; affects neurological function, autophagy and apoptosis of hippocampal neurons in AD [427]; controls development of osteoarthritis in chondrocytes [428]; suppresses myofibroblast activation in systemic sclerosis [429]; regulates the p53 signaling pathway in myoblast differentiation [430]; regulates postmenopausal osteoporosis [431].
hsa-miR-101-3p [UACAGUACUGUGAUAACUGAA]	Suppresses HER2-positive BC [432], HCC [433,434], glioblastoma [435], endometrial carcinoma [436], NSCLC [437], renal cell carcinoma [438] and melanoma [439]; regulates cancer proliferation [440]; regulates mitochondrial metabolic function [440]; induces vascular endothelial cell dysfunction [441]; regulates osteogenesis [442].
hsa-miR-30d-5p [UGUAAACAUCCCCGACUGGAAG]	Suppresses gallbladder carcinoma [443], rectal cancer [444], colon cancer [445], prostate cancer [446], ESCC [447], renal carcinoma [448], PC [449], HCC [450], THCA [451], LSCC [452] and NSCLC [453,454,455].
hsa-miR-378a-3p [ACUGGACUUGGAGUCAGAAGGC]	Controls metabolism, muscle differentiation/regeneration and angiogenesis [456]; suppresses glioblastoma [457] and HCC [458]; protects against intestinal injury [459]; modulates keratinocytes cell cycle arrest in psoriasis keratinocytes [460].
hsa-miR-191-5p [CAACGGAAUCCCAAAAGCAGCUG]	Inhibits replication of human immunodeficiency virus type 1 (HIV-1) [461].
hsa-miR-10a-5p [UACCCUGUAGAUCCGAAUUUGUG]	Inhibits osteogenic differentiation [462]; inhibits keratinocyte proliferation in atopic dermatitis [463]; reduces IL-6-induced cartilage cell ferroptosis [464]; regulates BDNF expression in follicular fluid [465]; suppresses renal cell carcinoma [466]; mitigates Ca^2+^ entry in T cells through gut bacterial metabolite urolithin [467]; enhances viability and migration of human umbilical vein endothelial cells [468].
hsa-let-7b-5p [UGAGGUAGUAGGUUGUGUGGUU]	Promotes protein processing in endoplasmic reticulum in acute pulmonary embolism [469]; promotes angiogenesis [470]; inhibits proliferation of leukemia [471]; inhibits proliferation of leukemia THP-1 Cells [471].
hsa-miR-200a-3p [UAACACUGUCUGGUAACGAUGU]	Prevents MPP^+^-induced apoptotic cell death [472].
hsa-miR-186-5p [CAAAGAAUUCUCCUUUUGGGCU]	Promotes apoptosis [473] and suppresses CRC [474].
hsa-miR-320a [CUCCCCUCCGCCUUCUCUUCCCGGUUCUUCCCGGAGUCGGGAAAAGCUG]	Suppresses CRC [475,476], glioblastoma [477], GC [478], salivary adenoid cystic carcinoma [479] and CML [480]; targets genes in lithium response in bipolar disorder [481]; regulates fibrotic process in interstitial lung disease of systemic sclerosis [482]; regulates cell proliferation and apoptosis in multiple myeloma [483]; regulates erythroid differentiation [484]; controls glucagon expression [485]; regulates cell proliferation and apoptosis in multiple myeloma [483]; stimulates endometrial stromal cell migration during preimplantation embryo stage [486]; improves skeletal muscle mitochondrial metabolism [487].
hsa-miR-181b-5p [AACAUUCAUUGCUGUCGGUGGGU]	Involved in Ang II-induced phenotypic transformation of smooth muscle cells in hypertension [488]; suppresses starvation-induced cardiomyocyte autophagy [488]; improves anti-tumor cytotoxic T cell response in B cells of CLL [489]; inhibits trophoblast cell migration and invasion in multiple abnormal trophoblast invasion [490]; modulates cell migratory proteins in endometrial stromal cells [491]; suppresses the progression of epilepsy [492]; suppresses gallbladder carcinoma [493].
hsa-miR-30e-5p [UGUAAACAUCCUUGACUGGAAG]	Regulates angiogenesis, apoptosis, cell differentiation, oxidative stress and hypoxia [494]; regulates autophagy and apoptosis in contrast-induced acute kidney injury [495]; suppresses NSCLC [496] and squamous cell carcinoma of the head and neck [497]; enhances innate immune responses [498]; suppresses cancer cell adhesion, migration and invasion, and considered as a potential target for curbing metastatic spread in P53-deficient tumors [499].
hsa-miR-103a-3p [AGCAGCAUUGUACAGGGCUAUGA]	Regulates BDNF expression in follicular fluid [465]; suppresses PC [500]; aggravates renal cell carcinoma [501]; regulates Wnt signaling pathway in colorectal carcinoma [502].
hsa-miR-182-5p [UUUGGCAAUGGUAGAACUCACACU]	Mediates downregulation of BRCA1, impacting DNA repair and sensitivity to PARP inhibitors [503]; suppresses CRC [504], renal cell carcinoma [505], bladder cancer [506] and prostate cancer [507]; acts as glial cell line-derived neurotrophic factor GDNF mimics dopaminergic midbrain neurons [508]; associates with renal cancer cell mitotic arrest [509].
hsa-miR-151a-3p [CUAGACUGAAGCUCCUUGAGG]	Enhances slug-dependent angiogenesis and regulates multiple functions in the lung, such as cell growth, motility, partial EMT and angiogenesis [510].
hsa-miR-335-5p [UCAAGAGCAAUAACGAAAAAUGU]	Regulates bone homeostasis [511]; suppresses GC [512], uterine leiomyoma [513] and ESCC [514]; regulates cardiac mesoderm and progenitor cell differentiation [515].
hsa-miR-25-3p [CAUUGCACUUGUCUCGGUCUGA]	Suppresses hepatocytes [516] and regulates osteoblast differentiation of human aortic valve interstitial cells [517].
hsa-let-7g-5p [UGAGGUAGUAGUUUGUACAGUU]	Suppresses epithelial ovarian cancer [517] and glioblastoma [518]; and alleviates murine collagen-induced arthritis by inhibiting Th17 cell differentiation [519].
hsa-miR-200c-3p [UAAUACUGCCGGGUAAUGAUGGA]	Suppresses prostate carcinoma [520], renal cell carcinoma [521] and epithelial ovarian cancer [522]; regulates integrin-mediated cell adhesion [523]; attenuates the tumor-infiltrating capacity of macrophages [524].
hsa-miR-30c-5p [UGUAAACAUCCUACACUCUCAGC]	Suppresses GC [525] and prostate cancer [526]; protects cells from hypoxia-reoxygenation-induced apoptosis and induces cell proliferation and anti-apoptotic and proliferative effects [527]; reduces cellular migration and pro-angiogenic gene expression in extracellular vesicle EV-recipient cells [528].
hsa-miR-429 [UAAUACUGUCUGGUAAAACCGU]	Inhibits cell proliferation and Ca^2+^ influx by pulmonary artery smooth muscle cells [285]; regulates hypoxia [529]; suppresses breast cancer [530], osteosarcoma [531], THCA, soft tissue sarcoma [532], cervical cancer [533], GC [534], diffuse large B-cell lymphoma [535], esophageal carcinoma [536], HCC [537,538], glioblastoma [539], NPC [540], PC cancer [541], THCA [542], OSCC [543] and renal cell carcinoma [544]; inhibits bone metastasis in breast cancer [545]; regulates the transition between HIF1A and HIF3A expression in human endothelial cells [546].
hsa-miR-99b-5p [CACCCGUAGAACCGACCUUGCG]	Suppresses primary myotubes [547], epidermal keratinocytes and cervical cancer cells [548].
hsa-miR-29a-3p [UAGCACCAUCUGAAAUCGGUUA]	Modulates CYP2C19 in human liver cells [549]; suppresses cell proliferation [550]; activates hepatic stellate cells, which moderate their profibrogenic phenotype, supporting the use of miR-29a agonists for treating liver fibrosis [551]; regulates tumorigenicity and TME development [552]; involved in the progression of HCC, which elucidates its potential theragnostic implications [552]; suppresses GC [553] and renal cell carcinoma by regulating E2F1 expression by long non-coding RNA H19 [554]; induces TNFα in endothelial dysfunction [555]; mediates tumor immune infiltration in breast invasive carcinoma [556]; acts as a protective factor for fibrogenesis in gluteal muscle contracture [557]; regulates osteoblast differentiation and peri-implant osseointegration [558]; promotes intestinal epithelial apoptosis in ulcerative colitis [559]; regulates and restores endothelial function in normal people and cardiometabolic disorders, respectively [560]; regulates peripheral glucocorticoid receptor signaling [561]; suppresses CRC [562], gliomas [563], head and neck squamous cell carcinoma [564], prostate cancer [565], GC [553], PTC [566], lung cancer [567], cervical cancer [568] and endometrial cancer [569]; enhances the radiosensitivity of OSCC cells [570]; modulates ALDH5A1 and SLC22A7 in human liver cells [571].
hsa-miR-30b-5p [UGUAAACAUCCUACACUCAGCU]	Suppresses HCC, which is sponged by long non-coding RNA HNF1A-AS1 oncogene [572]; inhibits GC cell migration [572]; regulates lipid metabolism [573]; involved in vascular smooth muscle cell differentiation [574]; involved in homocysteine-induced apoptosis in human coronary artery endothelial cells [575]; inhibits osteoblast differentiation [576]; inhibits proliferation and promotes apoptosis of medulloblastoma cells [577]; controls adverse effects of non-small cell lung cancer NSCLC radiotherapy [578]; mediates ferroptosis of trophoblasts, which is involved in the pathogenesis of preeclampsia [579]; suppresses expression of B-cell activating factor mRNA primary in Sjögren’s syndrome [580].
hsa-miR-19b-3p [UGUGCAAAUCCAUGCAAAACUGA]	Suppresses cell mobility [581]; involved in proliferation, apoptosis and cycle of SH-SY5Y cells [582]; regulates neuropathic pain and neuroinflammation [583]; regulates cell cycle in CRC [584]; induces endothelial dysfunction and decreases lung injury, inflammation and permeability and improved hemodynamics [585]; regulates skeletal muscle anabolism [586]; regulates apoptosis in THCA [587]; interacts with environmental factors, such as maternal stress during pregnancy, neonatal jaundice and family psychiatric history, to impact risk of ASD [588]; stimulates cardiomyocyte apoptosis [589].

All sequences were retrieved from https://mirbase.org/ (accessed on 22 April 2022). The top 10 miRNAs in both colostrum and mature milk are highlighted in green. Abbreviations: AD, Alzheimer’s disease; ASD, autism spectrum disorder; ATG, autophagy related; BDNF, brain-derived neurotrophic factor; ESCC, esophageal squamous cell carcinoma; MSCs, mesenchymal stem cells; DPMSCs, dental pulp-derived MSCs; T_reg_, regulatory T cells; ccRCC, clear cell renal cell carcinoma; NSCLC, non-small cell lung cancer; IGF, insulin-like growth factor; FGFR3, fibroblast growth factor receptor 3; GC, gastric cancer; GHR, growth hormone receptor; SOCS-7, suppressor of cytokine signaling-7; EMT, epithelial-to-mesenchymal transition; HBP4, hyaluronan binding protein 4; HIF, hypoxia-inducible factor; JAK2, janus kinase 2; ALL, acute lymphoblastic leukemia; ADH4, alcohol dehydrogenases; BC, breast cancer; LSCC, lung squamous cell carcinoma; TGF, transforming growth factor; TNBC, triple negative BC; THCA, thyroid cancer; SNRPB, small nuclear ribonucleoprotein-associated protein B; PC, pancreatic cancer; PTC, papillary thyroid cancer; PASMCs, pulmonary artery smooth muscle cells; AML, acute myeloid leukemia; HCC, hepatocellular carcinoma; CRC, colorectal cancer; CML, chronic myeloid leukemia; HDAC4, histone deacetylase 4; KIR, killer immunoglobulin-like receptor; LAMB3, laminin subunit beta 3; MS, multiple sclerosis; MAP4K4, mitogen-activated protein kinase 4; NPC, nasopharyngeal carcinoma; OSCC, oral squamous cell carcinoma; PCOS, polycystic ovary syndrome; RA, rheumatoid arthritis.

**Table 4:** List of studies that investigated the epigenetic effects of breastfeeding on different physiological and pathological functions in infants and mothers.

Disease/Condition	Type of Study	Target(s)	Study Criteria and Participants	Main Findings	Reference
Cancer	Case-control	The *p53* gene (the guardian of the genome)	In archived tumor blocks from 803 cases, the promoter methylation of the p16 gene in connection to breastfeeding was examined	The *p53* gene promoter was nearly three times more likely to be methylated in premenopausal women who had never been breastfed	[695]
Metabolism and growth	Cross-sectional	*LEP* gene	120 Dutch children (50 girls) were included with average age of 1.4 years	Children who were breastfed for at least 1 to 3 months had lower *LEP* promoter methylation in white blood cells and higher serum levels of leptin than children who were never breastfed	[727]
	Prospective observational cohort	*RXRA* and *LEP* genes	The effects of breastfeeding duration on infant growth and methylation in obesity-related genes of buccal cells (*n* = 101) were assessed	At 12 months, breastfeeding duration was associated with epigenetic changes in *RXRA* and *LEP* genes, as well as infant biometry and growth	[734]
	Cohort	*LEP* gene	23 CpGs in the *LEP* gene in 297 samples of 10-year-old and 16 CpGs in 305 samples of 18-year-old were tested for association with breastfeeding duration	Breastfeeding length is associated with *LEP* methylation at 10 years of age and BMI trajectory; despite the small sample size, *LEP* DNA methylation is related to BMI trajectories throughout childhood	[733]
	Cohort	The protein coding genes *FDFT1* and *SNX25*, as well as the ncRNA gene *LINC00840*	A comprehensive Epigenome-Wide Association Study to identify associations between breastfeeding and DNA methylation patterns in childhood (at birth, 10, 18 and 26 years) was performed. Breastfeeding durations of >3 months and >6 months, as well as exclusive breastfeeding durations of >3 months, were used to categorize the feeding.	In 10-year-old children who were breastfed for more than three months, a substantial differentially methylated region covering the gene FDFT1 was discovered	[735]
BMI and weight	Cohort	A total of 2 CpG sites in boys (NREP and IL16) and 13 CpG sites in girls (ATP6V0A1, DHX15/PPARGC1A, LINC00398/ALOX5AP, FAM238C, miR-21, SNAPC3, NATP/NAT2, CUX1, TRAPPC9, OSBPL1A, ZNF185, FAM84A, PDPK1) were investigated	The study comprised 15,454 pregnancies, with 15,589 known fetuses, 14,901 of whom were alive at one year. A total of 12,761 children were available for study after twins (*n* = 201) and children missing anthropometric measurements or age information were removed. The kids were tracked for more than two decades	CpG sites were shown to be enriched in miRNAs and critical pathways (AMPK signaling, insulin signaling and endocytosis). When compared to no breastfeeding, DNA methylation variation corresponding to 3 to 5 months of exclusive breastfeeding was linked to lower BMI growth in the first 6 years of life	[740]
Lung diseases and asthma	Cohort	This gene is linked to asthma risk	Blood samples were collected from 245 females at age 18 years randomly selected for methylation analysis from a birth cohort (*n* = 1456)	The number of weeks of breastfeeding had minor impacts on methylation of the interleukin-4 receptor gene’s relevant CpG island	[730]
Neurological disorder	Cohort	*DRD4* gene	The data came from a large population-based triple B pregnancy cohort study (*n* = 844) that included thorough information on maternal alcohol intake throughout pregnancy and in the early postpartum period. The methylation of the DRD4 promoter DNA was investigated	The methylation of a *DRD4* (a key dopamine receptor) in cheek cells was higher in eight-week-old children whose moms drank moderate amounts of alcohol during breastfeeding compared to those who did not drink	[742]
	Cohort (bioinformatics study)	Many genes, particularly those involved in oxytocin signaling pathway	Investigating the association of breastfeeding and DNA methylation in the peripheral blood cells of 37 children aged 9 months to 4 years	In response to breastfeeding, the oxytocin signaling pathway serves a unique role as a possible activator of coordinated epigenetic alterations in genes essential to CNS function	[751]
Immunity and allergy	Cross-sectional	*TLR1* gene	In 57 adult adults, DNA methylation at two locations in the promoter of the TLR1 gene, as well as the relationship between DNA methylation of the TLR1 gene and illness susceptibility, were studied	The promoter of the TLR1 gene showed a considerable reduction in DNA methylation. There was no link discovered between DNA methylation and illness vulnerability	[746]
